# Polylactide Perspectives in Biomedicine: From Novel Synthesis to the Application Performance

**DOI:** 10.3390/pharmaceutics14081673

**Published:** 2022-08-11

**Authors:** Carmen Moya-Lopez, Joaquín González-Fuentes, Iván Bravo, David Chapron, Patrice Bourson, Carlos Alonso-Moreno, Daniel Hermida-Merino

**Affiliations:** 1 Laboratoire Matériaux Optiques Photonique et Systèmes (LMOPS), CentraleSupélec, Université de Lorraine, 57000 Metz, France; 2 Centro Regional de Investigaciones Biomédicas (CRIB), 02008 Albacete, Spain; 3 Facultad de Farmacia de Albacete, Universidad de Castilla-La Mancha, 02008 Albacete, Spain; 4 Unidad NanoCRIB, Centro Regional de Investigaciones Biomédicas, 02008 Albacete, Spain; 5 DUBBLE@ESRF BP CS40220, 38043 Grenoble, France; 6 Departamento de Física Aplicada, CINBIO, Lagoas-Marcosende Campus, Universidade de Vigo, 36310 Vigo, Spain

**Keywords:** polylactide, stereocomplex, biomedicine, processing conditions, tailored pharmaceutical treatments, personalised medicine

## Abstract

The incessant developments in the pharmaceutical and biomedical fields, particularly, customised solutions for specific diseases with targeted therapeutic treatments, require the design of multicomponent materials with multifunctional capabilities. Biodegradable polymers offer a variety of tailored physicochemical properties minimising health adverse side effects at a low price and weight, which are ideal to design matrices for hybrid materials. PLAs emerge as an ideal candidate to develop novel materials as are endowed withcombined ambivalent performance parameters. The state-of-the-art of use of PLA-based materials aimed at pharmaceutical and biomedical applications is reviewed, with an emphasis on the correlation between the synthesis and the processing conditions that define the nanostructure generated, with the final performance studies typically conducted with either therapeutic agents by in vitro and/or in vivo experiments or biomedical devices.

## 1. Introduction

Biomaterials are natural or engineered substances that interact with components of living systems that can be exploited for a medical purpose, either as therapeutic or diagnostic agents [1]. The development of novel, customised solutions urged by society to minimise detrimental invasive side effects involves complex multifunctional compounds that feature ambivalent properties. Likewise, the molecular engineered design should consider the processing steps required to generate the final material as well as the mechanism of application to attain a high performance of the targeted activities. Particularly, metals are typically used when mechanical strength or electrical conductivity is required, whilst ceramics exhibit a high compressive strength and relative chemical inertness and polymers possess a great potential due to the chemical flexibility that endows them with a wide range of physical and mechanical properties, as well as activities through their functionalisation [2]. The use of biopolymers as agents for medical applications dates back thousands of years, when, in ancient India and Egypt, wounds were sutured by using natural polymers such as catgut and silk. Likewise, naturally occurring polymers, such as polysaccharides and proteins, are abundantly available and have been widely used in biomedicine ranging from wound dressing to arterial and skin grafts. However, its application in the field of medicine is limited due to the risk of infections, antigenicity, and batch-to-batch variability [3]. In contrast, the application of synthetic polymers in medicine was reported more recently during the Second World War, when Nicholas Harold used poly(methyl methacrylate) (PMMA) as an artificial corneal substitute [4]. Subsequently, several biostable (non-degradable under physiological conditions) synthetic polymers were employed in the biomedical field, such as polyethylene terephthalate (PET) for vascular grafts, polydimethylsiloxane (PDMS) for breast implants, and polyethylene (PE) for hip joint replacements [3], due to their mass production at an industrial scale that endows them with a low cost, batch-to-batch reproducibility, and flexibility for performing function-specific applications. In addition, hydrolysable polymers such as polylactic acid (PLA) were considered valuable for degradable surgical implants to avoid a subsequent clinical intervention for removing the medical implant [5]. Since then, the development of biodegradable (under physiological conditions) polymers, such as polyesters, poly-ester-urethanes, polycarbonates, etc., for biomedical applications, such as bone grafts, sutures, and 3D scaffolds, and for pharmaceutical applications such as drug delivery systems or polymer therapeutics has increased exponentially due to their tunable degradation properties, the ease of their processing and administration, as well as their chemical and biological properties that resemble numerous biological tissues [6,7].

The design of novel polymeric materials must fulfil the environmental and societal demands to diminish the carbon footprint required to adopt manufacturing strategies that meet the European policies such as “A European Strategy for Plastics in a Circular Economy”, which was launched in 2018 to address how plastics are designed, used, and recycled [8]. Likewise, the European Parliament has recently recognised the potential role of bioplastics and compostable plastics in the circular economy and sustainability [9,10]. Consequently, bio-based plastics synthesised from renewable resources such as PLA are promising environmentally friendly candidates for the development of biomedical and pharmaceutical applications whilst contributing to the circular economy.

PLA is a compostable polymer derived from corn sugar, potato, and sugar cane [11,12] whose promising physicochemical properties are comparable to those of petroleum-based polymers, such as PE, polypropylene, polystyrene, polycarbonate, and PET [13]. PLA is a semicrystalline polymer that hydrolyses in physiological media, yielding lactic acid, a non-toxic component that is eliminated via the Krebs cycle as water and carbon dioxide [14]. The biocompatibility, biodegradability, and resorbability characteristics of PLA have promoted its use in the biomedical field for a wide range of applications (suture threads, bone fixation screws, drug delivery systems, etc.), offering an alternative to conventional biocompatible materials such as metals and ceramics [15]. In addition, the ability to tailor the mechanical, thermal, and degradation properties of PLA derivatives due to the range of afforded nanostructures depending on the chemical architecture and processing conditions allows for designing personalised medical solutions. Indeed, the novel available synthetic PLA approaches to generate multiblock copolymers as well as the advent of current processing technologies broaden its suitability to advance into the customisation of the generated end-products to minimise the adverse side effects. In particular, several highly cutting-edge PLA-based therapeutic applications have recently attained the clinical phase, such as drug-eluting stents [16,17,18,19] as well as personalised pharmaceutical agents that were designed from an interdisciplinary approach to avoid the serendipitous progress, emphasising the beneficial interaction between the materials and the biomedical fields. Likewise, the frequent dismissed PLA nanostructure of the designed biomedical solutions hamper the systematic advance by correlating the structure–property relationship of the system with the application performance. Several reviews about PLA have been published covering a wide range of topics, such as the PLA synthesis [20,21], the physicochemical and mechanical properties of PLA [22], the crystallisation and structure–properties relationship of PLA [23], the characteristics of the promising stereocomplex PLA phase [24,25], and PLA applications in widespread fields [11,15,26,27,28,29,30]. Herein, the state-of-the-art of PLA-based biomedical applications is reviewed from a global approach by highlighting the interconnections among the architectural designing parameters with the desired applications, with an emphasis on the stereocomplex phase of PLA.

## 2. Poly-(lactic Acid) (PLA)

Polylactic acid is a poly-α-hydroxy acid synthesised from lactic acid (LA; 2-hydroxypropanoic acid) which exists in two optically active stereoisomers, namely, L-LA and D-LA (S and R in absolute configuration, respectively) [31]. Approximately 90% of the total lactic acid produced worldwide is made by bacterial fermentation, which offers advantages in both the utilisation of a renewable source and the production of optically pure L- and D-lactic acid, depending on the strain selected (the chemical synthesis of lactic acid always results in a racemic mixture). The dehydrated cyclic dimer of lactic acid is commonly called lactide (3,6-dimethyl-1,4-dioxane-2,5-dione). Lactide exists in three different forms due to the presence of two asymmetric carbon atoms in the molecule: L-lactide, D-lactide, and meso-lactide. In addition, a racemate of D-lactide and L-lactide exists as rac-lactide [31]. The chirality of PLA adds new functionality to PLA applications such as the specific recognition and interaction with chiral molecules (drugs, proteins, DNA, etc.) [32].

**Figure 1:**
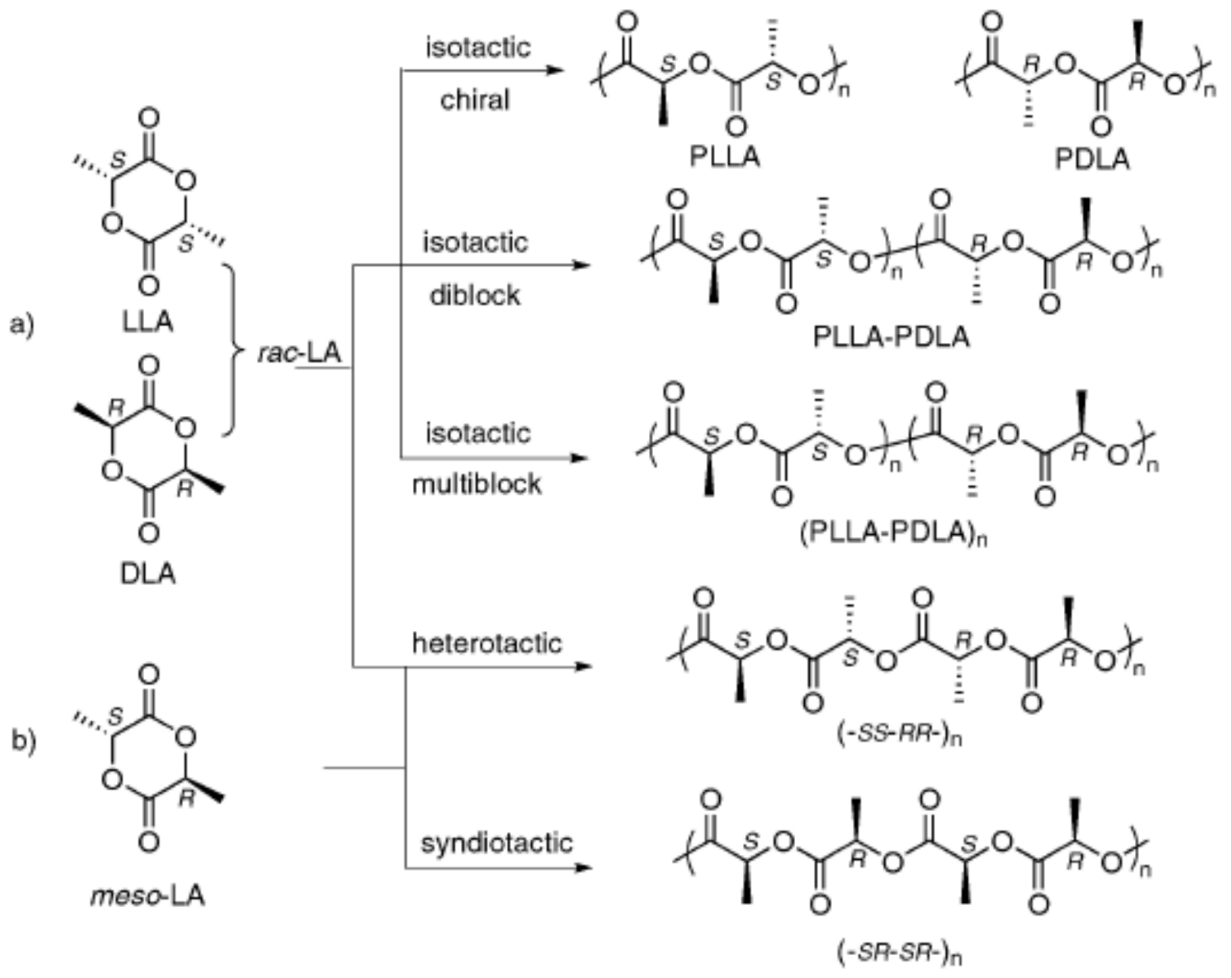
PLA microstructures. (**a**) Rac-Lactide, (**b**) meso-lactide. Reproduced with permission from Ref. [33]. Copyright 2010, The Royal Society of Chemistry.

The polymerisation of optically pure L- and D-lactide yields isotactic homopolymers of Poly–(L-lactide) (PLLA) and Poly–(D-Lactide) (PDLA), respectively (Figure 1). Both PLLA and PDLA are semicrystalline polymers, showing a melting temperature (T_m_) around 170 °C [20,33] and a thermal degradation temperature around 200 °C [34]. The PLA derivative crystallinity as well as their melting and glass transition temperatures (T_g_) usually decrease with the diminishing optical purity of the lactate units [22,35]. PLLA polymers with a D-lactide content lower than 10% tend to be crystalline (or PDLA with L-lactide content), whilst homopolymers with a lower optical purity are amorphous [36]. The random insertion during the polymerisation of D- and L-LA units of both rac- and meso-lactide monomers generates an atactic polymer, Rac-PLA, which is completely amorphous. Moreover, syndiotactic PLA is obtained when D- and L-lactic acid units are placed alternatively along the chain, whereas a heterotactic chain architecture is attained when D- and L-lactide units are inserted alternatively on the polymer chain [37]. Syndiotactic PLA is a semicrystalline polymer exhibiting higher T_c_ than isotactic PLA but lower T_m_, whilst heterotactic PLA is amorphous [38]. Furthermore, the one-pot sequential addition polymerisation method [39] of D- and L-lactide monomers yields stereo-block copolymers with blocks of opposite chirality, featuring melting temperatures 50 °C higher than those of isotactic homopolymers (220 °C) [25].

PLA displays different crystalline phases (α, α′, β, γ) established by the chain architecture and the specific crystallisation mechanism or thermo-mechanical history imposed during its processing, which define the properties of the final product [40]. The α-phase is the more stable PLA homocrystal structure that corresponds to an orthorhombic unit cell in which the helices are packed in a hexagonal fashion, containing two antiparallel chains per unit cell. The α-phase is normally obtained when isotactic PLLA or PDLA are crystallised from the melt above 130 °C or by crystallisation from the solution, characterised by a melting temperature of around 170 °C [41,42]. The α′-phase (or δ-phase) is the disordered form of the α-phase that is generated either from crystallisation from the melt at temperatures below 110 °C or by cold-crystallisation after quenching PLA to the glassy state. The α’-phase is also organised in the orthorhombic crystallographic unit cell; however, it contains two parallel helices per unit cell, which increase the lattice parameters when compared to the ordered α phase. A mixture of α- and α’-phases is obtained when PLA is crystallised between 110 °C and 130 °C, although the α’-phase recrystallises into the α-phase when heated near the T_m_ (150–160 °C) [41]. Moreover, the β-phase is developed by stretching PLA fibres in the α-phase at a high temperature (130–140 °C) and high draw ratios as well as by casting thin films from the solution [42,43]. The chain conformation of the β-phase is a threefold helix in a trigonal unit cell containing three chains per unit cell, and its T_m_ is ca. 10 °C lower than α-phase T_m_ [44,45]. In addition, the β-phase exhibits piezoelectricity that allows for the interchange of mechanical and electrical energy [45], broadening its potential applications. Finally, the γ-phase is produced by the epitaxial crystallisation of PLA on hexamethylbenzene, forming two antiparallel threefold helices in an orthorhombic unit cell [46].

Furthermore, a new crystal structure, the stereocomplex (SC) phase, is formed from the co-crystallisation of the two stereoisomers of PLA (PLLA and PDLA) that feature a trigonal unit cell comprised of six threefold helices per unit cell. The structural peculiarity of the SC phase, with the nearest neighbours of any stem being of a different polymeric chain, provides them easy access to the growth front for both enantiomeric species. In addition, the specific C-H···O-H hydrogen bonds within the crystal lattice that stabilise the structure [47] endow stereocomplexes with a higher melting point (220 °C) and degradation temperature (220–260 °C) [34]. The SC phase was first found by casting a mixed solution of both enantiomers [48], and since then, the SC phase has typically been obtained intentionally from the blend of both enantiomers in the solution (in an appropriate solvent such as dichloromethane or chloroform at room temperature or acetonitrile around boiling temperature [49]) or in the solid-state from the melt [24]. However, the SC crystallisation of the blended enantiomers diminishes for high-molecular-weight (HM_w_) PLA, and enantiomeric homocrystals (HC) in the α-phase are obtained instead [50]. Additionally, the critical M_w_ to exclusively obtain SC crystallisation is lower for blends obtained from the melt than those obtained from the solution [51], which hampers its industrial application. The optical purities of the polymers and the mixing ratio of the isomeric chains also affect the obtained ratio of SC-to-HC crystallites, and thus, the preparation of pure SC-PLA requires meticulous specific conditions [52]. SC crystallites can also be generated through the synthesis of block copolymers by the one-pot sequential monomer addition to a truly living polymerisation catalyst, which allows for the retention of the SC crystallisation in HM_w_ polymers [53,54].

The new synthetic approach affords a wide range of chain architectures that can be generated through different ratios of L- and D-Lactide monomers that offer the possibility to tailor the properties of the final polymeric product depending on the intended application. Furthermore, understanding the advantages and drawbacks of the different synthetic processing methods to obtain PLA is crucial to tailoring the foreseen applications. Moreover, since PLA still exhibits performance drawbacks such as low mechanical properties, a low thermal resistance, and a low hydrophobicity, which limit its applications in some biomedical fields, novel materials with unique properties can be obtained through the blend or copolymerisation of PLA with other biodegradable or non-biodegradable polymers, such as polyethylene (PE), polypropylene (PP), Polyhydroxhyalkanoates (PHA), PMMA, Poly(ethylene-*co*-vinyl acetate) (PEVA), etc. [13,55,56]. In addition, nanocomposites can be fabricated by mixing PLA with other complementary compounds such as silk [57], gelatin [58], collagen [59], tungsten disulfide [60,61,62], natural fibres (flax, jute, hemp,) [63], ceramics (ZnO, TiO_2_) [64,65], etc. to enhance their performance.

## 3. Synthesis of PLA

PLA was first synthesised by polycondensation by Théophile-Jules Pelouze in 1845. In 1932, Wallace Hume Carothers developed a novel synthetic method based on the ring-opening polymerisation (ROP) of the cycle lactide monomer to synthesise PLLA. ROP was later patented by Du Pont in 1954 to synthesise vinyl fluoride (U.S. Patent No. 2674632, 1954). However, HM_w_ PLA by ROP on an industrial scale was only attained by the mid-1990s [31].

The lactic acid monomer can be converted to PLA through a polycondensation process by the reaction of the hydroxyl (–OH) and carboxylic acid (–COOH) groups with the removal of the detrimental byproducts such as water. Generally, catalysts are added to polymerisation to increase the reaction rate. The removal of water, enhanced under vacuum pressure, is critical to producing HM_w_ polymers due to the increased viscosity of the reaction mixture as the reaction proceeds. However, side reactions, such as transesterification, can also occur during the polycondensation of lactic acid, resulting in the formation of ring structures of different sizes, such as lactides. Transesterification reactions lower the overall M_w_ and the stereocontrol over the chain architecture, decreasing the physical properties of the PLA afforded as well as reducing the reaction yield [35]. The HM_w_ PLA is mainly synthesised by ROP due to the accurate chemical control in terms of molecular weight, polydispersity, polymer chain-ends, and tacticity. Moreover, ROP requires relatively mild conditions (130 °C) when compared to polycondensation (180–200 °C) [35,66]. Three reaction mechanisms have been proposed for ROP of lactide: anionic, cationic, and coordination-insertion mechanisms. In both anionic and cationic polymerisations, a monomer-activation mechanism occurs first, which permits the catalyst to be independent of the propagating polymer and can thus be easily removed as the polymerisation finishes. However, undesirable side and racemisation reactions are likely to occur due to the highly activated monomers. On the contrary, coordination-insertion polymerisation attains HM_w_ PLA with higher control over the M_w_ distribution [14,35]. Metal complexes of several metals have been widely employed as the catalysts for the ROP of lactides [35], of which the most studied are stannous 2-ethylhexanoate [Sn(Oct)_2_], aluminium isopropoxide [Al(O i-Pr)_3_], and zinc(II) lactate [Zn(Lact)_2_] [66]. Sn(Oct)_2_, is the catalyst utilised for the industrial synthesis of PLA, largely due to its approval by the FDA for use in medical (<20 ppm [67]) and food applications. Moreover, lauryl alcohol (1-dodecanol) is usually added as an initiator [35].

**Figure 2:**
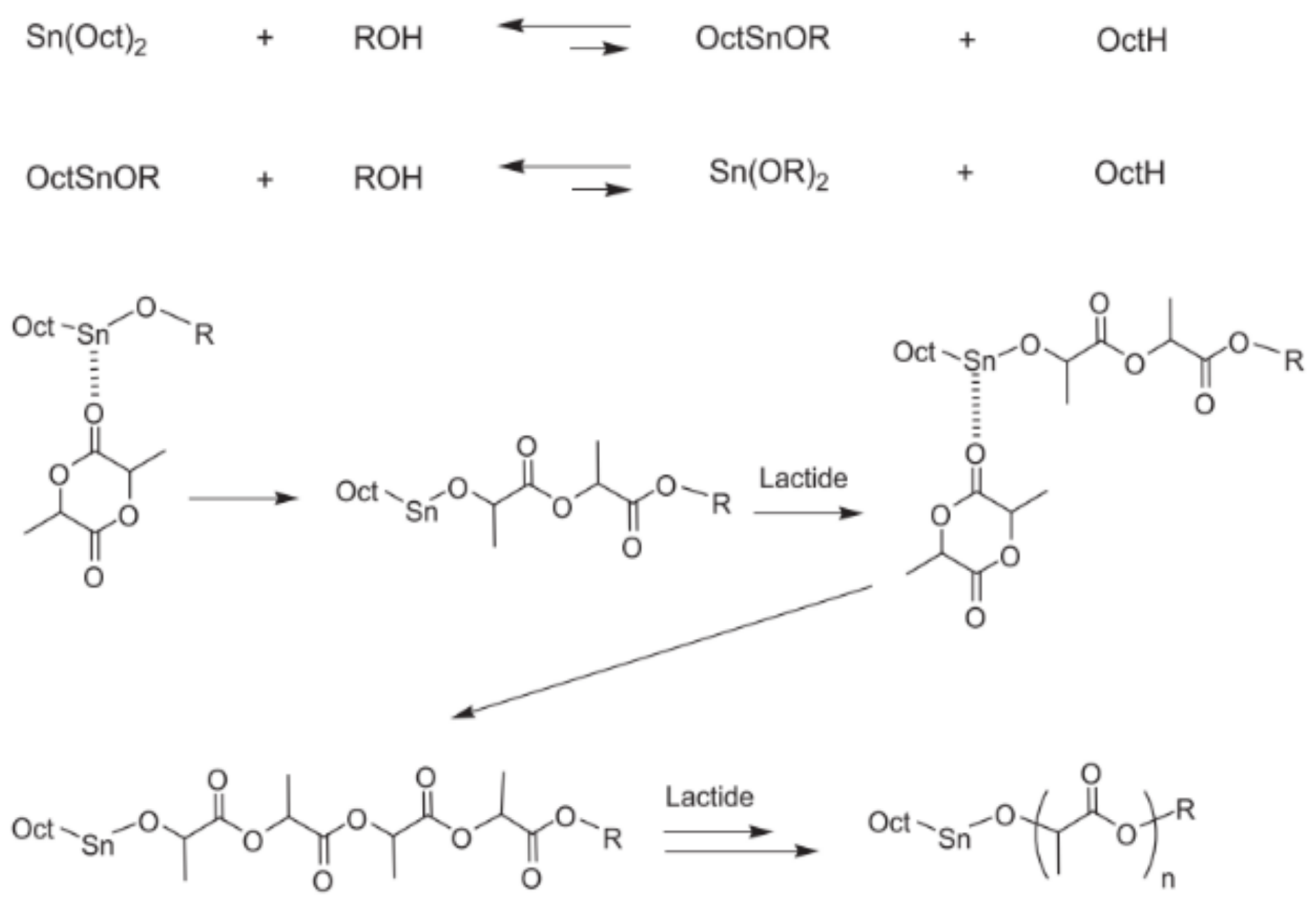
ROP of lactide by Sn(Oct)_2_ by the coordination-insertion mechanism. Reproduced with permission from Ref. [35]. Copyright 2015, The Royal Society of Chemistry.

**Scheme 1:**
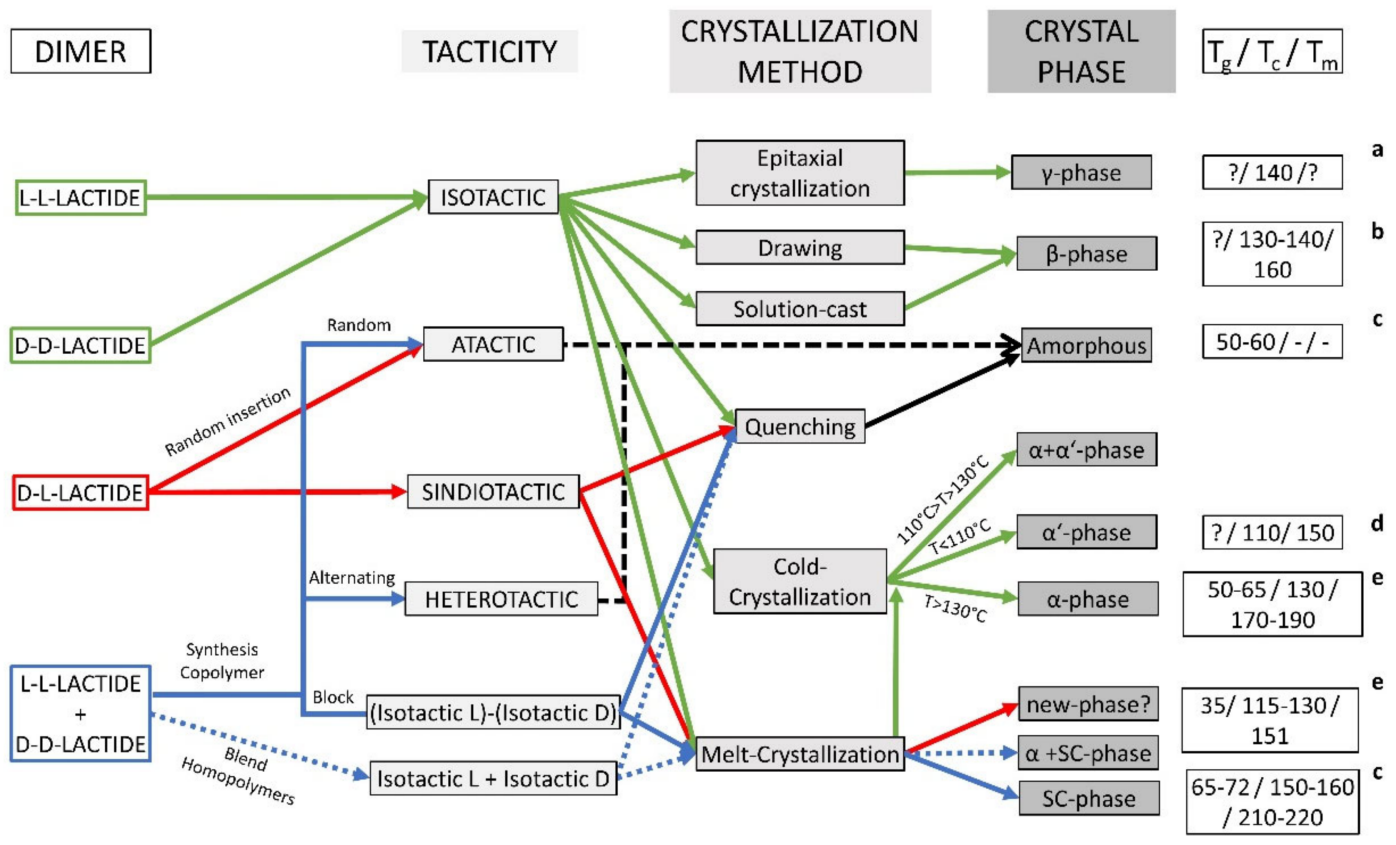
Overview of the morphology attained by the different PLA derivatives depending on the processing conditions. Dimer refers to lactide dimer starting materials for ROP polymerizations. T_g_: glass transition temperature; T_c_: crystallisation temperature; T_m_: melting temperature; ?: No information found. ^a^ [46]; ^b^[74]; ^c^[24]; ^d^ [41]; ^e^ [38].

The polymerisation is induced by a coordination-insertion three-step mechanism supported by the catalyst [66], which was first formulated in 1971 by Dittrich and Schulz [14] (Figure 2). Firstly, Sn(Oct)_2_ reacts with the lauryl alcohol to form a tin alkoxide. Subsequently, the exocyclic carbonyl oxygen of lactide temporarily coordinates with the tin atom of the catalyst in the alkoxide form. The formed coordination system enhances the nucleophilicity of the alkoxide part of the initiator as well as the electrophilicity of the lactide carbonyl group that enables the reaction to each other. Finally, the acyl-oxygen bond of lactide is disrupted, and the generated linear chain of the lactide turns into the alkoxide part of the catalyst, promoting the coordination with a new lactide molecule and, thus, the polymerisation propagation [35]. Finally, the active metal-alkoxide bond is hydrolysed as the monomer is entirely consumed, and the formation of a hydroxyl end-group occurs [66]. In the last stage of the propagation step, as the monomer concentration becomes significantly lower (~80%), both intra- and inter-molecular transesterification reactions occur, and the molecular weight distribution increases. However, the degrees of racemisation and chain scrambling achieved by metal complexes that follow coordination-insertion mechanisms are notably lower than those obtained by anionic or cationic catalysis [35]. The microstructure of the final polymers depends both on the initial monomers added to the reaction mixture (Scheme 1) and the catalyst stereocontrol. The control exerted by the catalyst over the nanostructure of the PLA, particularly to synthesise the PLA stereo block copolymers of HM_w_, is essential to tailor the properties of the final product, and the synthesis of novel catalysts, particularly metal-based catalysts, for polymerisation by the coordination mechanism has attracted much attention since the pioneering work of Kleine et al. in the 1950s [66,68,69]. However, several drawbacks to controlling the synthesis of stereoblock HM_w_ PLA have emerged since then, such as the decrease in the living character of the catalyst due to the increase in the reaction heterogeneity [70], the detrimental side reactions due to the multiple nuclearities exhibited by the catalysts [71], as well as the long reaction time required to achieve the desired architectures and molecular weight [14]. Recently, novel catalysts that fulfil the synthetic requirements whilst exhibiting low toxicity for the application of the PLA derivatives in the biomedical and pharmaceutical fields were just attained [54,72,73], which offer the possibility to design multiblock copolymers simultaneously featuring the PLA stereoblock to attain higher physicochemical properties with other complementary blocks to tackle the PLA limitations.

## 4. PLA Processing

Once synthesised, PLA is usually manipulated into its final shape by the use of different processing techniques that apply diverse thermomechanical histories. Melt processing is a three-step process generally used to transform PLA into different commodity products at an industrial scale [75]. Firstly, the polymer is melted to subsequently be moulded into the desired shape, which is then generally cooled to stabilise its dimensions. Widespread industrial techniques such as extrusion and injection moulding are the two most common melt-based processes for manufacturing thermoplastic polymers. Novel common additive manufacturing technologies with promising personalised biomedical applications such as fused deposition modelling follow a melt-based process [76]. Likewise, the tailoring of T_m_ of PLA is crucial for the performance of applications employing melt-based processing techniques, as the process temperature must be above T_m_ to form a homogeneous melt but low enough to minimise thermal degradation [77]. In addition, the cooling rate during the third step will influence the properties of the final product, which determine the crystallisation conditions that dictate the crystallinity degree and crystalline phase. Particularly, quenching PLA from the melt at a high cooling rate (>500 °C/min, such as during injection moulding) will result in a highly amorphous polymer [78], whilst semicrystalline PLA is obtained when the cooling rate is reduced (>30 °C/min [79]). The α-form is usually developed from PLLA or PDLA during typical melt processing; however, a mixture of the α and α′ phases is obtained when the cooling rate is higher than 2 °C/min since the α-form has very slow crystallisation kinetics (pure α-phase is obtained at slower rates) [80]. Furthermore, post-production treatments such as annealing can be implemented to increase the thermal stability and mechanical properties of the final product. The α-phase develops completely from a melt-crystallised material exhibiting a mixture of α and α’when annealing at 140 °C for 1 h [80]. Additionally, obtaining only the SC phase from the melt from the equimolar blend of PLLA and PDLA enantiomers can be achieved by restricted thermodynamic conditions such as relatively low cooling rates (20 °C/min) to avoid phase separation or from low-molecular-weight enantiomers (~20 kDa) [50] and isothermal crystallisation at temperatures above the T_m_ of homocrystals (~175 °C) [51], as well as by melt-spinning under high-tensile-stress conditions [81] (Scheme 1). However, PLA materials crystallised in the stereocomplex phase have not reached the market yet [82].

Three-dimensional (3D) printing is an additive manufacturing technology with the unique ability to produce personalised objects with complex designs at reduced costs and a high-resolution precision [83,84]. They have already reached the market in the biomedical field such as the manufacturing of 3D scaffolds for studying the response of particular tissues to different stimuli [85]. In particular, PLA is the most-used polymer for 3D printing since the slow crystallisation rate compared to that of polyolefins (i-PP or PET) avoids warping between layers [86]. Classical processing methods such as particulate leaching, gas foaming, or solvent-casting were used for the tuning of the internal architecture of 3D scaffolds due to their adequacy for replacing tissues with a high regenerative capacity. However, 3D printing enables the required control over the scaffold architecture for fewer regenerative tissues, such as tendons or nerves [87]. Likewise, PLA 3D-printed scaffolds have already been investigated for bone [88,89,90,91,92,93], neural [94], and musculoskeletal soft [95] tissue engineering. In addition, PLA nanofibres have also been used as part of a fibrous bioink for the 3D printing of a meniscus construct to study the proliferation of human adipose-derived stem cells that provide a higher cell proliferation and metabolic activity [95]. Recently, the assessment of the PLA scaffold geometry effect on the orthopaedic applications [96] revealed that the presence of hydroxyapatite (HA) in the scaffold efficiently enables mineralisation as well as induces the crystallisation of PLA after being 3D printed, whilst PLA without HA remained amorphous. The presence or absence of crystalline domains within the 3D-printed PLA scaffold will invariably influence the hydrolysis degradation rate, which is crucial to controlling the optimum performance of the biomaterial. However, the usual lack of structural study for most of the reported 3D-printed PLA scaffolds for biomedical applications impedes the determination of the relationship between the processing conditions, the crystalline structure, and the biomedical performance.

Furthermore, in the biomedical industry, electrospinning has been considered a promising method to fabricate polymer nanofibres due to its simplicity and the cost-effectiveness of the technique. Electrospinning, unlike drying spinning, which relies on mechanical extrusion, uses the electrostatic force to spin the solution into fibres [78]. The fibres thus obtained have a nanometric diameter, producing materials with a high area/volume ratio, a high flexibility, and superior mechanical properties compared with other material formats [97]. Electrospun PLA usually exhibits either an amorphous structure or a semicrystalline structure, although with a very low crystallinity (~10%), due to the rapid solidification of the fibres during the process, which entails post-processing thermal treatment between the T_g_ and the T_m_ to enhance the crystallinity by cold-crystallisation in the α-phase [98,99]. Recently, the straightforward fabrication of PLA electrospun fibres exhibiting the β-phase without further post-processing treatment was achieved [100], facilitating the development of PLA-based devices with piezoelectric properties for potential biomedical applications, as will be further discussed [101]. Furthermore, PLA fibres in the SC phase were also generated from the electrospinning of PLLA/PDLA blends, resulting in more uniform fibres [102]; however, an annealing post-processing step is usually required to obtain crystalline structures [103,104]. In addition, the tensile strength and Young’s modulus was found to be modulated by varying the spinning method, i.e., either by melt-spun, solution-spun, and/or as-spun [103]. Moreover, plasma protein adsorption was also investigated on solution cast films of PEG-PLA and compared to the SC phase formed by the PEG-PLLA/PEG-PDLA blend. The absorption of both albumin and fibrinogen was higher on the SC films than on the homopolymer counterparts. The SC crystallisation was revealed to hamper the migration of the PEG to the surface of the film, prolonging protein adsorption and cell attachment over a longer period [105]. The large efforts dedicated to electrospun nanofibres in the biomedical field raise the possibility to mimic the extracellular matrix, since the human tissues and organs are formed by nanofibrous scaffolds [106,107]. However, industrial-scale production of PLA nano-fibres has not been achieved yet due to the low throughput of the technique and the requirement of specific solvents [78]. Nevertheless, PLA nanofibres are a topic in continuous research due to their crucial role in several biomedical applications such as bone regeneration [108,109], drug delivery systems [110], and wound dressing [111].

**Figure 3:**
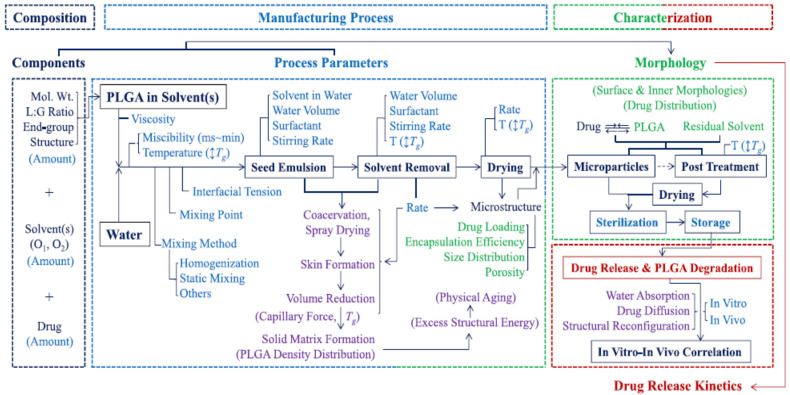
Manufacturing process of PLGA microparticles by emulsion methods and the parameters affecting the final properties of the formulation. Reprinted with permission from Ref. [112]. Copyright 2021, Elsevier.

Furthermore, PLA can also be processed into nanoparticles to generate drug delivery systems in which the drug release rate can be controlled by varying parameters such as the processing method, the microstructure of the starting polymer, or its concentration in the organic solvent [112]. However, contradictory results are usually found when relating certain processing parameters to the final nanoparticle release kinetics [113,114,115], and hence, further studies based on the structure–property relationship are required to understand the mechanistic process occurring during nanoparticles formation to design drug nanocarriers with tailored drug release profiles [112] (Figure 3). Furthermore, the methods for preparing nanoformulations most commonly utilised at the laboratory scale, such as nanoprecipitation or emulsification-solvent evaporation, lack reproducibility between batches. However, novel approaches such as supercritical technology, electrospraying, or premix membrane emulsification have emerged as methods that are better adapted for industrial production, enabling the scale-up process of nanoparticles [116].

In addition, PLA is generally employed to afford hydrogels, as is capable of absorbing a large amount of water that can be programmed to be expanded or shrunk due to external condition changes [117]. Hydrogels are aqueous dispersions that solidify by the decrease in polymer solubility in response to different physical and/or chemical stimuli—typically, pH or temperature—that are used to control the drug delivery systems [118]. PLA is usually copolymerised with a hydrophilic polymer to form associative micelles that constitute the gel in which the nanostructure and rheological properties can be tuned by varying the stereochemistry of the PLA [119,120]. Moreover, PLA can also be part of hydrogel materials as a mechanically reinforcing and/or drug-eluting component [58,121,122]. PLA-based hydrogel studies are less common than PLA-based nanoparticle studies. However, a deeper structural characterisation is usually accomplished.

## 5. PLA Properties

When designing a PLA-based material for biomedical applications, physicochemical properties such as the mechanical and thermal behaviour as well as the degradation degree under physiological conditions must be considered for the chain architecture synthesis together with the subsequent impact of the processing methods to correlate the structure–property relationship with the targeted application.

### 5.1. Mechanical Properties

The mechanical properties of PLA are mainly associated with the crystalline phase and crystallinity degree [40], which must be tailored by controlling the stereocontrol exerted during the synthesis as well as the subjected thermal history and pre-treatment conditions. The mechanical properties of PLA range from soft and elastic (amorphous PLA) to stiff and high-strength (semicrystalline PLA) [22], and thus, semicrystalline PLA is preferred when robust mechanical properties are required.

Amorphous PLA is typically obtained from rac-PLA as well as from isotactic PLA quenched from the melt. Semicrystalline PLA has a tensile strength of 60 MPa, a flexural strength of 100 MPa, and an elongation at break of about 4%, whilst amorphous PLA has lower tensile and flexural strengths (40 MPa and 84 MPa, respectively) and a higher elongation at break (7.5%) [75]. The mechanical properties are also dependent on M_w_ [22]—in particular, semicrystalline PLA is more affected compared to amorphous PLA [75], as the chain entanglements, which influence the crystallinity and hence the mechanical properties, increase as the Mw increases [123]. Likewise, the tensile strength increases by a factor of 10 when M_w_ is increased only by a factor of 4 [124]. Semicrystalline PLA presents higher mechanical properties than a few relevant commodity polymers, such as PS, i-PP, and PET, but an inferior thermal resistance [125]. PLA is thus more susceptible to thermal degradation during processing, lowering its M_w_ and hence its final mechanical properties (M_w_ of PLA decreased by 14–40% after injection moulding [126]).

The mechanical properties of PLA as a function of crystal polymorphisms have also been investigated, although mainly for the α- and α’-forms due to their abundant availability under industrial processing conditions. Likewise, the heat deflection temperature of injected-moulded PLA materials was found to increase linearly with increasing crystallinity above ca. 35–40% as well as with the annealing temperature increase, which could be explained by the rise of the α/α’ ratio [80,127]. Furthermore, PLLA materials crystallised in the α-phase by annealing after quenching feature a larger Young;s modulus, tensile strength, and storage modulus as well as a lower elongation-at-break and water vapour permeability when compared to those crystallised in the α′-phase [127,128], which may be attributed to the tighter molecular packing of PLA chains in the α-phase [128]. However, PLA crystallised from the melt showed a decrease in tensile strength, elongation at break, and unnotched impact strength as the temperature of crystallisation increased (isothermally analysed between 100 °C and 130 °C) [129]. Moreover, the Young’s modulus increased as the temperature increased, except for the PLA crystallised at 130 °C (α-phase), which is associated with the formation of an aggregate between the amorphous and crystal region due to the formation of fewer nuclei [129]. The discrepancy between results may be attributed to the difference in spherulite density dependent on thermal treatment (annealing after quenching vs. crystallisation from the melt) [130]. In addition, the tensile modulus and tensile strength of PLA also increase for the β phase compared to the α-phase [43]. Similarly, tensile properties such as tensile strength, Young’s modulus, elongation at break, and storage modulus are also improved by the stereocomplexation of PLA [131]. 

### 5.2. Degradation

Degradation is a crucial feature to be considered when designing PLA biomedical materials with tailored properties. The main factors that affect the mechanism and rate of PLA degradation depend on the polymer characteristics such as the molecular weight, crystallinity, and shape and/or size as well as the physicochemical parameters related to the surrounding medium such as temperature, pH, and/or enzymes [132].

Commonly, PLA degradation is a multistep process that is initiated through a hydrolytic process followed by enzymatic action, since biotic attack only occurs when PLA reduces its M_w_ [133]. However, in the human body, only the hydrolytic process occurs, followed by the degradation of lactic acid in the Krebs cycle to finally yield water and oxygen. PLA hydrolysis follows the ester moiety cleavage from the polymeric backbone chain, decreasing the M_w_:

–[C(RR′)COO–] + H_2_O → –C(RR′)COOH + –C(RR′)COH

Moreover, PLA hydrolysis is an autocatalytic process, as the chain scission increases the concentration of carboxylic acid end groups in the degradation medium, which possesses a catalytic action on the process (Equation (1)) [134].

d[COOH]/dt = k[COOH][H_2_O][E] (1)

where [COOH], [H_2_O], and [E] are the concentrations of the carboxylic groups, water, and ester groups in the medium, respectively. The hydrolytic degradation of PLA typically occurs in a stepwise fashion. Firstly, water is diffused into the material that triggers the later hydrolysis of the chains in the amorphous region; subsequently, the diminution of M_w_ proceeds as a result of the hydrolytic cleavage of ester bonds and the formation of water-soluble compounds; finally, the hydrolysis of the crystalline phase occurs [135].

- Polymer factors

Hydrolytic degradation is inversely proportional to M_w_ below 4 × 10^4^ g/mol due to the higher mobility of chains and the larger density of hydroxyl groups, which enhance the probability of water-soluble oligomers formation, thus catalysing PLA degradation [136]. However, the hydrolysis rate is no longer dependent on M_w_ above 8 × 10^4^ g/mol since other parameters, such as total crystallinity, have a greater effect on hydrolysis [137].

Semicrystalline PLA is more resistant to hydrolytic degradation than amorphous polymeric chains, including the amorphous regions between crystalline parts. [132]. The hydrolysis of amorphous PLA showed a weight decrease of about 14% after 18 weeks, whilst semicrystalline PLA lost a similar weight after 20 months (at pH 3.4 and 37 °C) [135]. However, accelerated hydrolysis in the early stages has been observed with the increase in the initial polymer crystallinity (X_c_) at pH 7.4 [138]. The higher density of hydrophilic terminal groups in the amorphous regions between the inter-crystalline areas accelerates the initial hydrolytic degradation of crystallised PLA regions by enhancing the diffusion of water molecules into the bulk material. Likewise, films formed by mainly amorphous PLA undergo hydrolytic degradation with a nearly steady hydrolysis rate constant (k_h_) throughout the process, whilst films composed of predominantly semicrystalline PLA are subjected to a two-step hydrolytic degradation, initiated by a higher k_h_ stage, followed by a smaller k_h_. Moreover, the degradation rate of the SC crystalline phase is smaller than that of the α-phase [139].

In addition, degradation is faster as the thickness of the object is increased [140]. The size is related to the hydrolytic degradation as a result of the solubility of the oligomers close to the surface, which are drained into the solution as the ageing time increases. The solubilised oligomers within the matrix remain entrapped and contribute to the autocatalytic effect (Equation (1)). Likewise, the main PLA degradation mechanism was found to be dependent on several size regimes: bulk erosion for a material thickness lower than 2 mm, core-accelerated erosion between 2 mm and 7.4 cm, and surface erosion for a thickness higher than 7.4 cm [132].

- Media factors

**Figure 4:**
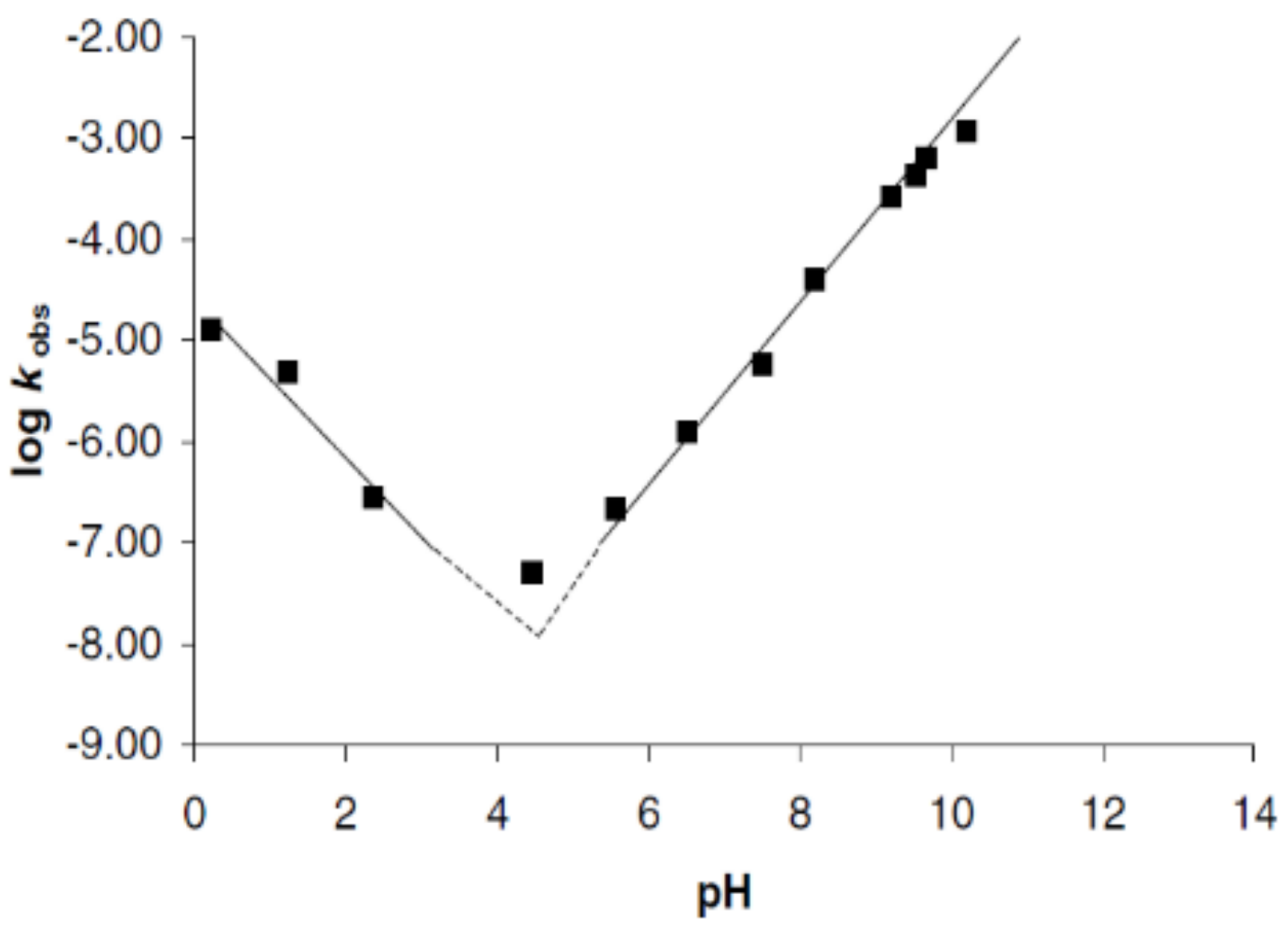
Dependence of the hydrolytic degradation constant (*k_obs_*) on pH. Reprinted with permission from Ref. [141]. Copyright 2001, Elsevier.

Furthermore, the main media factors that affect PLA hydrolysis are pH and temperature. Particularly, understanding the degradation kinetics dependence of PLA on pH is crucial to generating materials with tailored properties for biomedical applications due to the pH of the human body ranging from strongly acidic to almost neutral. Recent efforts have been focused on elucidating the effect of pH in the PLA hydrolysis mechanism of degradation. The pH dependence of the k_h_ was found to be described by the general rate constant equation (Equation (2)) [141]:

k_h_ = k_o_ + k_H_ [H^+^] + k_OH_ [OH^−^] (2)

where k_o_ is the first-order rate constant degradation in water, and k_H_ and k_OH_ are the second-order rate constants catalysed by protons and hydroxyl ions, respectively. A pH regime dependence described the hydrolysis mechanism specifically hydroxyl-catalysed above pH 5 (Figure 4, the slope is ~1 in the plot of log k_h_ against pH), and specific proton catalysis occurs at pH values below pH 4 (Figure 4 slope ~−1), whilst k_h_ is minimal at a pH value around 4.5 [141,142]. Likewise, the dependence of the hydrolysis mechanism on pH was proved for degradation under acidic conditions, which occurs through the split of the last lactic acid monomer after the nucleophilic attack by water [141,142,143]. However, the mechanism of PLA degradation in neutral or alkaline media is controversial, and the decomposition route is believed to follow either an intramolecular transesterification with the formation of lactide from the two end units of the oligomer [141] or a random ester cleavage degradation mechanism [142].

In addition, the effect of temperature on the hydrolytic degradation (T_h_) was found to be differentiated into three thermal regimes related to the chain mobility: T_h_ < T_g_, T_g_ ≤ T_h_ < T_m_, and T_m_ ≤ T_h_. The hydrolytic degradation rate increases when T_h_ is higher than T_g_ in contrast to T_h_ being lower than T_g_ as a consequence of the higher chain mobility. Likewise, if T_h_ exceeds T_m_, crystalline regions melt and disappear, and thus, the hydrolytic degradation in the melt occurs homogeneously, similarly to racemic PLA. However, when T_h_ is lower than T_m_, the hydrolysis degradation causes a heterogeneous M_w_ reduction as a result of the lower water diffusion. Indeed, the activation energy of the hydrolytic degradation increases from 50.9 kJ/mol for the thermal range T_m_ ≤ T_h_, to 83 kJ/mol for T_h_ < T_g_, whilst an intermediate activation energy of 69.9 kJ/mol is found for the temperature regime T_g_ ≤ T_h_ < T_m_ [132].

Moreover, the rate of degradation is also dependent on the magnitude and type of the applied stress that is crucial to designing biomedical materials employed in physiological conditions. Materials subjected to a static load experience a higher rate of degradation, which is further increased when a dynamic load is applied [144].

## 6. Medical Applications

PLA and its copolymers are currently used at the clinical level in different pharmaceutical applications, such as dermal fillers and drug delivery systems (DDS), as well as in biomedical applications such as sutures or tissue engineering (TE) [7,145,146,147]. The first biomedical application of PLA at the clinical level, namely, the suture Vycril^®^ composed of glycolide and lactide components, dates from 1974. Since then, several sutures composed of different ratios of glycolide and lactide have reached the market, such as Polysorb^®^, Radik^®^, or XLG^®^ [7]. Moreover, Decapeptyl SR was the first pharmaceutical product based on PLGA microparticles approved in Europe in 1996 for the treatment of prostate cancer. Thereafter, more than 15 microparticle PLA-based products have been approved and marketed to control drug delivery [145] and as a soft-tissue augmentation agent [146,147].

The recent trends of PLA in pharmaceutical and biomedical research will be addressed in the following section. The pharmaceutical section comprises the applications related to drug delivery such as nanoparticles, whilst the biomedical section includes the applications related to tissue reparation and the diffuse limit between both fields.

### 6.1. Pharmaceutical Applications

#### 6.1.1. Nanoparticles

PLA is among the biocompatible polymers most frequently used as DDS due to the ease of tuning the drug release profile from PLA formulations by modifying different architectural parameters such as the polymer molecular weight, stereochemical composition, or polymer crystallinity. Nowadays, several PLA-based microparticle formulations have successfully reached the market [145]; however, microsized DDS feature inherent drawbacks to traversing biological barriers, as well as their rapid clearance after systemic administration, limiting the application of microparticles to subcutaneous and intramuscular injections [148]. In contrast, nanosized DDS increase the systemic circulation time and facilitate tissue penetration as well as sterilisation via filtration [148]. PLA nanoparticles have been designed for the treatment of several pathologies such as cancer [149,150], Chagas disease [151], Alzheimer disease [152], or insulin-dependent diabetes [153]. The surfaces of PLA nanoparticles have been functionalised with antibodies [154] or cell-penetrating peptides [155,156] as a synthetic approach to target specific tissues or cells in order to increase the therapeutic effect and decrease side effects. Moreover, the attachment of PLA nanoparticles to cells exhibiting blood barrier brain migratory properties (T lymphocyte) has also been proposed as a novel approach to drug delivery into the brain [157].

However, the unusual establishment of the correlation of the structure–property relationship of the PLA-based nanoparticles after formulation, such as the T_g_, the crystallinity, or the physical ageing, makes it difficult to understand the drug release mechanism from a nanoscopic scale (Table 1). Particularly, the rearrangement of polymer chains during physical ageing, which typically occurs during the storage step and depends on the T_g_, may increase water absorption, leading to an initial burst release [158]. The control and analysis of the polymeric physicochemical parameters of the generated formulations are crucial to designing a specific degradation rate considering also the influence of the performance conditions on the drug-release mechanism: drug diffusion through water-filled pores, diffusion through the polymer matrix, osmotic pumping, or surface and bulk erosion [148].

#### 6.1.2. Hydrogels

PLA-based hydrogels (Table 2) that are formed through different external stimuli have already reached the clinical level. Particularly, Atridox^®^ is a doxycycline formulation used for chronic periodontitis containing PLA and N-methyl-2-pyrrolidone mixtures that solidifies to a wax-like consistency upon contact with gingival crevicular fluids. Furthermore, OncoGel^®^ is a paclitaxel formulation used for local tumour treatment based on the PLGA-PEG-PLGA triblock copolymer that exhibits a sol-gel transition at body temperature [160]. The fabrication of PLA-hydrogels usually requires copolymerisation with other polymers such as polyethylene glycol (PEG), polyurethane and polyglycolic acid [161], as well as the blend with collagen and gelatin [58,59] to increase the hydrophilic character. Moreover, different physicochemical and mechanical properties, such as the drug release rate, can be modulated by varying the size of the PEG block, the polymer concentration, as well as the stereoisomery of the PLA [162,163]. Likewise, a series of stereocomplexed PLA pentablock hydrogels were synthesised with a tailored microstructure by varying the stereostructure, crystallisation, and stereocomplexation, and their physical properties were thoroughly characterised. The PLA hydrogels featuring symmetric pentablock copolymers exhibited higher a gel-sol transition temperature, a higher storage modulus, and a slower biodegradation and drug release compared to asymmetric pentablocks [164]. Recently, the synthesis of hydrophobic polymers, in particular PLA, to produce thermohydrogels has been achieved without the use of a solvent, which enhances thermohydrogels’ biocompatibility [165]. Furthermore, a PLA-based hydrogel was designed that might be applied as a delivery vehicle with immediate release upon contact with reducing conditions, such as tumour microenvironments [166]. The polymer solution gelled between 32 °C and 40 °C due to the decrease in polymer solubility and collapsed upon exposure to strong reducing agents due to the cleavage of the disulphide bond incorporated in the polymer structure [166]. Likewise, a PLA-based hydrogel was recently designed for the treatment of chronic inflammatory diseases, such as rheumatoid arthritis, which fulfils an on-demand drug release depending on the severity of the disease by using nitric oxide (overproduced in inflammatory environments) as a hydrogel degradation agent [167]. PLA-based injectable hydrogels were also envisaged to be used for regenerative medicine requiring higher mechanical properties. Particularly, hydrogel composites comprising electrospun-PLA nanofibres and gelatin nanoparticles led to a 15-fold increase in the storage modulus without compromising the injectability [168]. Moreover, the incorporation of PLA nanoparticles into a gelatin hydrogel also leads to a 100-fold increase in the viscosity, without compromising the injectability [121]. Although the characterisation of the physicochemical properties of PLA-based hydrogels is generally conducted in a comprehensive way, the hydrogels should also be evaluated under physiological conditions, such as in vitro and in vivo studies, which is unusually accomplished (Table 2).

#### 6.1.3. Pharmaceuticals Design

**Figure 5:**
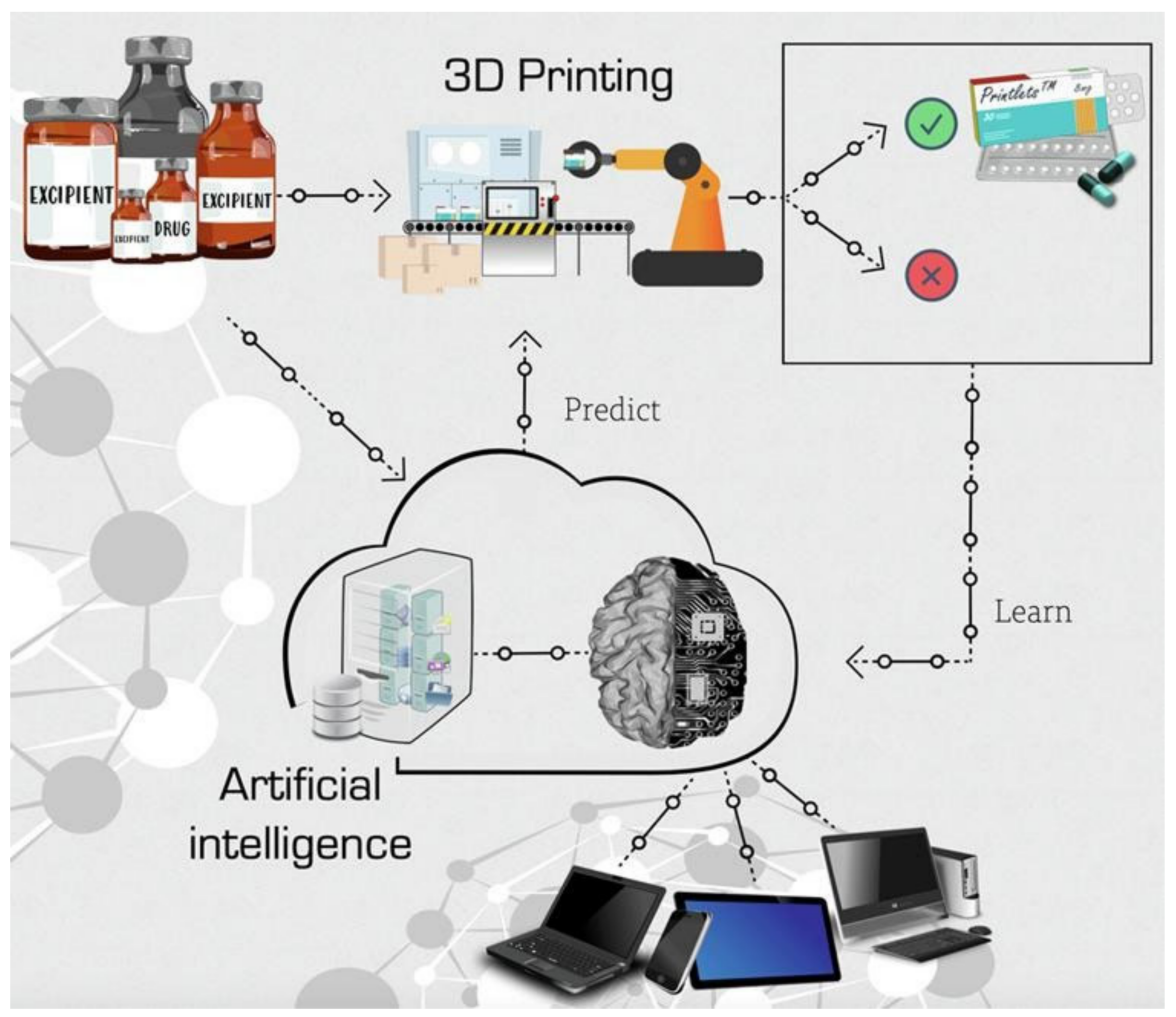
Machine learning approach for predicting the 3D printability of medicines (M3DISEEN). Reprinted with permission from Ref. [83]. Copyright 2020, Elsevier.

The application of 3D printers for the customisation and personalisation of pharmaceuticals is one of the most revolutionary and powerful tools in the last decades [171,172]. The traditional one-size-fits-all treatment approach in the healthcare industry is ineffective in up to 70% of patients, according to the National Health Service, due to the varied pharmacokinetic traits of different patients, creating the need to shift from mass production to personalised dosage medicine [173]. 3DP technologies have been used for the production of pharmaceutics for pre-clinical animal models due to the accuracy in producing dosage forms of appropriate geometry and size [174]. The Food and Drug Administration (FDA), in 2015, approved the first drug manufactured using 3DP technology, Spritam^®^ [175,176], opening the scope of oral drug delivery using 3DP. Likewise, a five-in-one dose combination polypill was manufactured by three-dimensional extrusion with two independently controlled and well-defined release profiles [177], simplifying the drug therapy and potentially improving the adherence of patients to the prescribed treatment. Additionally, artificial intelligence machine learning techniques have been developed to predict key 3DP fabrication parameters and advance the 3DP fabrication process, reducing the time, costs, and resources normally invested in formulation development [83] (Figure 5). The current variety of biomedical applications attained by PLA materials processed by 3D technology, such as the oral formulation of solid dosage forms [178] and subcutaneous implants for prolonged drug delivery [179] by fused deposition modelling, increases as the 3D techniques advance and the utilisation of 3DP is expanded in society.

#### 6.1.4. Chiral Drugs

**Figure 6:**
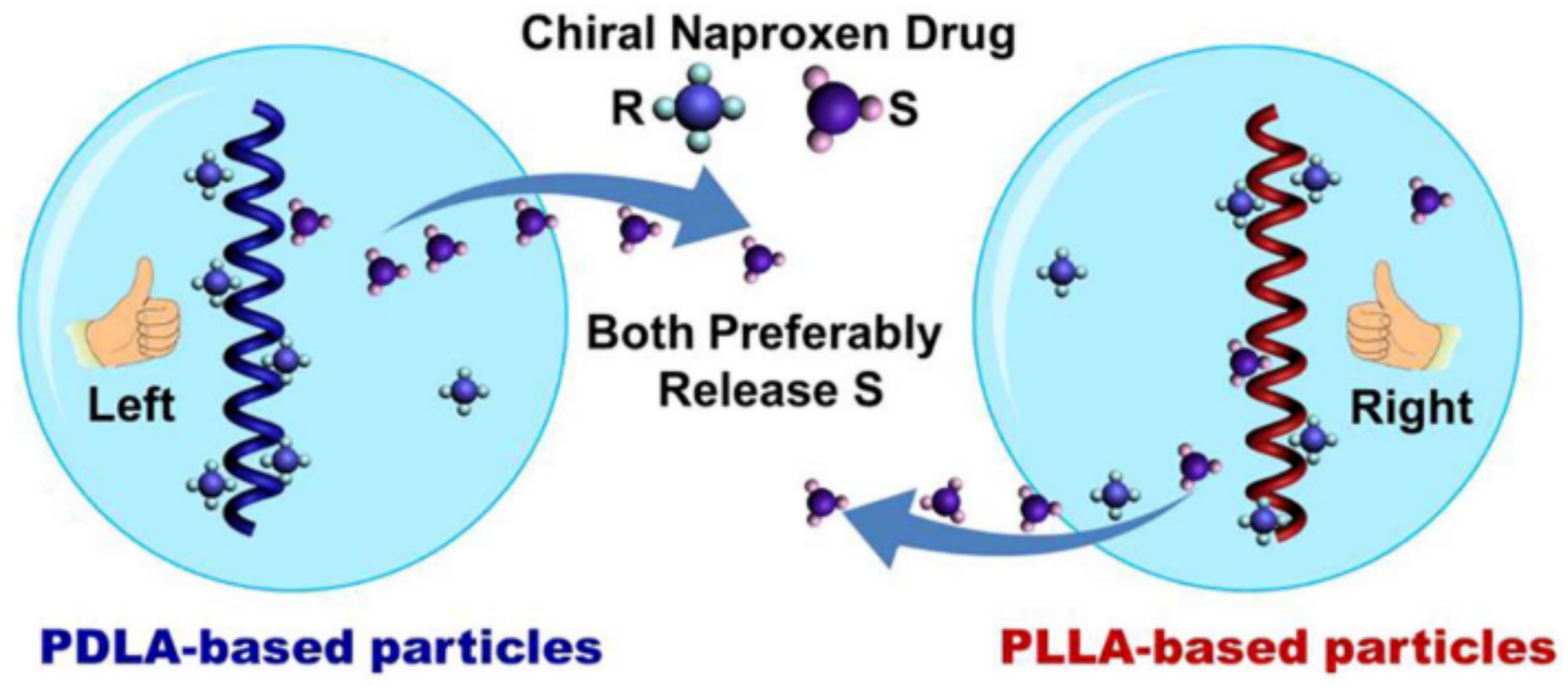
Polylactide-based chiral particles with an enantio-differentiating release ability. Reprinted with permission from Ref. [180]. Copyright 2018, Elsevier.

The development of chiral drugs has recently become important for the pharmaceutical industry, as, frequently, the enantiomers of a racemic drug have different physiological activities and pharmacokinetic profiles (e.g., R-Thalidomide is a sedative, S-Thalidomide leads to birth deformity). However, the vast majority of drugs used clinically are still employed as racemates, likely due to the difficulty in separating racemic compounds into enantiomerically pure isomers. The chirality of PLA enables its use for enantioselective drug delivery [32], as proved by the generation of chiral particles of PLLA and PDLA loaded with racemic naproxen, which preferably releases *S*-naproxen isomer in ethanol and chloroform for both PLLA and PDLA particles. The enantioselective release of *S*-naproxen was found to result from a different distribution of *R*- and *S*-naproxen within the particle caused by enantioselective interaction [180] (Figure 6). Furthermore, the drug release of the designed drug nanocarrier by chiral particles composed of two chiral helical polymers (PLA and the polymer based on chiral acetylenic (PCM)) revealed that polymer chains with a similar chirality (*S*-PCM/PLLA and *R*-PCM/PDLA) cooperated to release *R*-naproxen, while, for *S*-PCM/PDLA and *R*-PCM/PLLA, the particles preferably released *S*-naproxen [181]. In addition, hetero-stereocomplexes were reported to form between poly-D-lactide and L-configured proteins [182] such as insulin [183], somatostatin [184], or leuprolide [185]. Particularly, different factors such as the increase in the PDLA molecular weight or the Leuprolide/PDLA ratio increased the leuprolide release rate [186]. Furthermore, Leuprolide induced PDLA crystallisation in a less stable crystal phase (β-phase) that recrystallises into the α-phase upon further heating [187]. DNA-loaded PLA [188] as well as PLA-PEG [189,190] nanoparticles were also recently developed, where DNA acts as a nucleating agent due to the strong interactions between PLA/DNA molecules, promoting the crystallisation of PLA nanoparticles, which is primarily responsible for the sustained release of DNA. In addition, the DNA melting point shifted to a higher temperature in the PLA-DNA complex, suggesting the good protecting ability of the PLA matrix towards the incorporated DNA. Finally, the PLA-DNA complex exhibited a high transfection efficiency, which is a crucial step to ensuring the efficacy of the DNA molecule in being transcripted and translated. However, the PLGA nanoparticles exhibited higher gene transfection due to the higher DNA release [189]. Moreover, PLGA nanoparticles loaded with the correspondent antigen stimulated robust mucosal IgA immunity after intranasal administration for both parainfluenza virus [190] and tuberculosis [191].

#### 6.1.5. Antibacterial Applications

**Figure 7:**
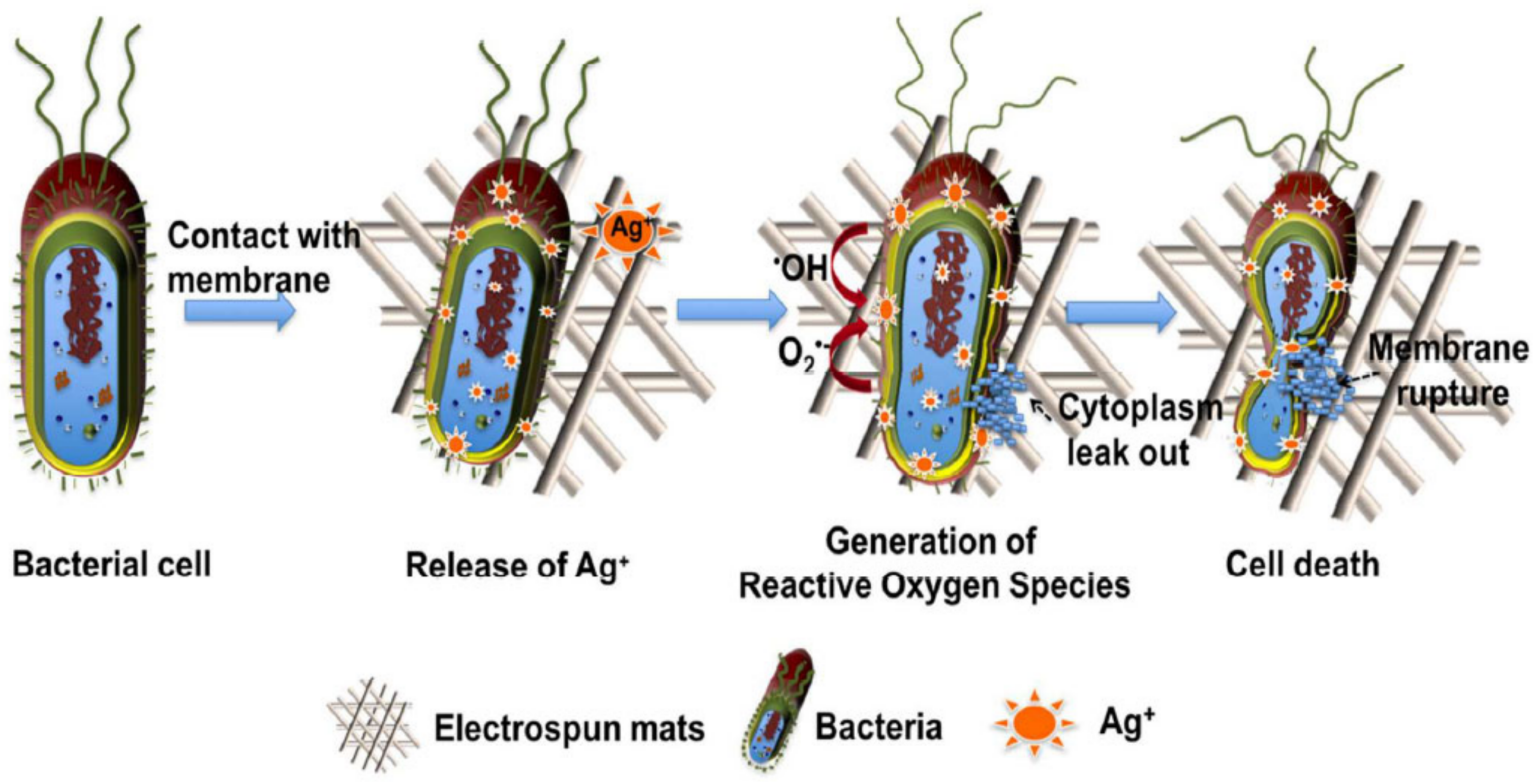
Schematic diagram of the release of Ag^+^ ions from AgNPs comprising PLA electrospun membranes exhibiting antibacterial properties. Reprinted with permission from Ref. [201]. Copyright 2017, American Chemical Society.

Bacterial infections originating from implants and medical devices are typically treated with antibiotics, and their misuse has promoted their resistance to drugs. Multidrug-resistant bacteria are one of the main threats to health and food security worldwide, according to the World Health Organisation, and alternative treatments mainly based on bacterial proliferation prevention need to be addressed [192]. Polymers usually do not exhibit intrinsic antibacterial properties, although antibacterial additives can be easily incorporated, such as silver nanoparticles [193,194], titanium dioxide [195], or essential oils, [196] to develop nanocomposites exhibiting antimicrobial activity. Moreover, antibacterial PLA filaments for 3DP that contain cooper particles are already commercially available (PLACTIVE AN1 Copper3D [197], PLActive Red [198]). Likewise, antibacterial 3D prostheses have been developed to minimise skin disorders related to microbial infections [199]. PLA-based nanocomposites exhibiting antibacterial characteristics can be manufactured in a wide variety of architectures due to the capability of PLA to be processed by different techniques, such as 3DP [200], electrospinning [201] (Figure 7), extrusion [202], spin coating [203], etc., which broadens its applicability in the biomedical field.

#### 6.1.6. Polymer Therapeutics

Polymer therapeutics is an alternative approach to conventional drug delivery systems which comprises polymer-drug/protein conjugates as well as polymers with a therapeutic effect in and of themselves (polymeric drugs). The lack of therapeutic activity featured by PLA has determined its conjugated use with drugs due to its higher stability, precise drug loading efficiency, and sustained drug release compared to drug encapsulation systems [204,205]. Several synthetic methods have been proposed to conjugate PLA into different drugs. Particularly, paclitaxel has been employed as an initiator for the ROP of rac-PLA through the reaction of the 2′OH of paclitaxel with lactide, ensuring the presence of paclitaxel at the chain termini [206]. In addition, paclitaxel has also been conjugated to rac-PLA throughout the PLA chain in a controlled way through an azide-alkyne click reaction that afforded the conjugate with a higher therapeutic effect than the free drug, as confirmed by in vitro cytotoxic analysis [207]. Antihypertensive drugs such as lisinopril have also been conjugated to PLA-PEG-PLA copolymers, followed by micelle formulation, exhibiting a more sustained release compared to physically loaded micelles [208]. Moreover, anti-inflammatory drugs such as indomethacin have also been covalently immobilised onto PLLA films as a potential coating for the metallic implants to avoid biofilm and blood clot formation [209]. However, the unusual nanostructure characterisation of the PLA component of the polymer–drug conjugate, which is typically based only on the molecular weight, morphological aspects, and thermal properties (T_g_, T_c_, T_m_, crystallinity), deters the understanding of the release kinetics [210] (Table 3).

### 6.2. Biomedical Applications

#### 6.2.1. Tissue Engineering and Scaffolds

Tissue engineering (TE) is one of the main strategies of regenerative medicine that consists of the regeneration of neotissues by applying 3D scaffolds for cell attachment and growth [211]. The designed scaffolds must fulfil different criteria with ambivalent properties such as biocompatibility and mechanical properties suitable for the intended application whilst supporting the normal functioning of cells and tissues. Polymers are the most popular materials for scaffold production due to their physicochemical characteristics and bioactivity [212]. Particularly, synthetic polymers have drawn significant attention as an alternative to natural polymers such as peptides, nucleic acid, and polysaccharides due to their price and the ease of their production [213]. Among the synthetic polymers, PLA, PGA, poly-(e-caprolactone) (PCL), polydioxane, and poly-(trimethylene carbonate) are currently the most extensively used for tissue engineering applications [213], and the specific polymer selection is based on the structure–property relationship required for the targeted application. Likewise, PCL features a high drug permeability and fewer acidic byproducts compared to other polyesters but a relatively slow degradation rate, limiting its usage to long-term applications. In contrast, PLA presents a faster degradation rate compared to PCL, although still relatively slow. However, long degradation times coupled with the high crystallinity of the remaining fragments might cause inflammatory reactions in the body. Likewise, PLA is used frequently as rac-PLA to diminish detrimental health effects, the latter being rapidly degraded without the formation of crystalline fragments [15]. Alternative approaches to circumvent the individual limitations of single polymers such as synthetic co-polymers, the combination with natural polymers, and/or scaffold surfaces functionalisation are frequently used to overcome the manifested issues such as hydrophilicity compatibility, a low cell attachment, and biodegradability concerns at the application conditions [212].

However, 3D scaffolds generated from PLA and its copolymers [212] have already been evaluated for a wide variety of regenerative applications with different tissues such as bone [214], nerve [215], tendon [216], and cartilage, which enlarges the applicability of conventional ceramic and metal solutions focussed on the orthopaedic field (Table 4). Particularly, polymers can be shaped into complex topologies for customised medical solutions with the advent of 3D printing technologies.

##### Bone Regeneration

Bone fracture is one of the more common injuries which is associated with treatment costs exceeding billions of dollars, societal productivity losses, and individual disability. Approximately 5 to 10% of fractured bones result in incomplete healing [226]. Currently, stainless steels- and titanium-based bone plates remain dominant in the internal fixation of bone fractures [227]. However, metallic implants usually lead to health complications, including bone atrophy and infection, and their subsequent removal can result in increased weakening and bone re-fracture due to the presence of the remaining screw holes. Additionally, the organisation and function of each bone are highly site-specific and related to its embryological origin [228].

In recent years, the use of biodegradable polymer plates for bone regeneration has garnered attention [15]. PLA and PGA are the biopolymers more commonly used due to the featured higher mechanical properties compared to other polyesters and the sufficiently low elastic modulus, avoiding the stress shielding caused by metallic implants [227]. PLA was proposed as a potential substitute for titanium plates to heal bone fractures 30 years ago, and several generations of PLA-based materials have been developed since then, especially for the treatment of maxillofacial fractures [217]. The third-generation materials composed of uncalcined hydroxyapatite and PLLA have reached the market (FIXSORB MX or OSTEOTRANS MX), and several clinical studies have been conducted, exhibiting promising results [217]. However, titanium plates remain in daily clinical use due to the persistence of drawbacks of the PLA properties—in particular, the slow degradation rate, which leads to a foreign body reaction and thus inflammation two years after implantation. The increase in the thickness of PLLA plates to match the strength of titanium plates increases the risk of exposure and patient discomfort [229], which might be improved by the fabrication of PLA plates in the SC phase to increase the strength, avoiding the thickness increase. In addition, a fourth PLA-based material generation was recently developed, which includes a small amount of PGA to overcome the slow degradation issue and its associated detrimental body inflammation [224,226,227]. PGA exhibits the highest initial mechanical properties but lessens the strength very rapidly in the body due to its hydrolytic instability, resulting in incomplete bone healing and a high local acid concentration, causing inflammation [230], which turns the copolymer poly (lactide-*co*-glycolide) (PLGA) into the best option for bone regeneration. PLGA mechanical properties and the degradation lifetime can be modified based on the PLA/PGA ratio (the lower the PLA/PGA ratio, the faster the PLGA is expected to degrade). Moreover, silk [214], hydroxyapatite [231], or collagen [59] can be included in the scaffold to increase the hydrophilicity, bone regeneration ability, and tensile strength of the composite, respectively.

Recently, PLA applicability in bone tissue repair has advanced to tackle characteristic limitations by the enhancement of the mechanical properties and the degradation rate and the optimisation of PLLA osteogenic activity [229]. Moreover, the optimisation of the 3D printing parameters of PLA/Hydroxyapatite composites for bone plates has recently been developed, correlating the processing conditions with the final mechanical properties of the composite [232].

##### Tendon Regeneration

PLA and, in particular, its copolymer with poly-glycolide (PLGA) have also been employed for other tissue applications such as tendon regeneration. Tendinopathies represent about 30% of tendon-related injuries [220], and spontaneous tendon healing results in problematic restitution due to the low cellular and hypo-vascular nature of tendon tissue [221]. Bio-polymeric 3D scaffolds provide a means to both heal tendon injuries and understand cell behaviour and differentiation in response to defined external biochemical and mechanical stimuli [222]. Particularly, a 2 cm partial resection of the Achilles tendon was repaired in rabbits using a composite scaffold composed of an outer part of knitted PLGA and an inner part of unwoven PGA fibres. The generated scaffold was incubated in adipose-derived stem cell culture and cell-seeded assemblies formed a neo-tendon presenting a histological structure similar to that of native tendon 45 weeks after implantation [216]. PLGA has also been employed for tendon regeneration applications as a nanocarrier to ensure the sustained and controlled delivery of human growth differentiation factor 5 [222], which is crucial for the expression of genes linked to the neo-tendon type. Similarly, stem cells with different origins were selected to understand the physiopathology of tendinopathy, in which PLGA was always used as a 3D scaffold [223,224]. Recently, the influence of certain production parameters to tailor the final nanofibre morphology, such as the alignment and the fibre size, were probed for an electrospun PLA scaffold on the differentiation of amniotic epithelial stem cells towards tenogenic lineage, indicating that highly aligned electrospun fibres [221] and smaller fibre diameters [220] enhanced cell differentiation and immunomodulation. Moreover, the mechanical properties of the electrospun scaffold were also influenced by the fibres alignment and its diameter; particularly, the scaffold featuring a higher diameter exhibited a lower Young’s modulus [220] and a higher fracture strain, whereas the alignment of the fibres increased the stress and strain values [221].

##### Nerve Regeneration

Peripheral nerve injuries are the most common injury types affecting the nervous system, and the posterior recovery of sensibility and motor functions is estimated to be only less than 3% and 25%, respectively. Peripheral nerve injuries can be repaired by surgery; however, the use of grafts becomes necessary when an important loss of physical substance occurs (>5 mm) [233]. Synthetic conduits of PLA/PLDLA, combined with longitudinal PLA filament scaffolds, were shown to enhance axon myelinisation in a rat sciatic nerve lesion model when compared to a silicone conduit [215]. In addition, the neural precursor cells, which are an interesting source of cells for neural tissue regeneration, generate a high number of detrimental reactive oxygen species during metabolism. Furthermore, the lactic acid degradation product released from PLA scaffolds has been shown to reduce the intracellular redox state that increases the proliferative capacity of the neural cell population [234].

##### Cartilage Regeneration

Similarly to tendons, cartilage tissue exhibits little ability to self-repair due to the limited vascularity. Likewise, the polymeric approaches were envisaged due to their versatility and the lack of current satisfactory solutions for cartilage tissue regeneration by other means [235,236]. Electrospun scaffolds based on PLA and gelatin loaded with the antioxidant resveratrol showed a high specific surface area, slow drug release, and thinner diameter to promote the repair of cartilage injury [237].

In addition, thermogels containing PEG-sc-PLA-Chol were evaluated as scaffolds for cartilage tissue engineering, in which the cholesterol block enhanced the mechanical properties, enlarged the pore size, and improved chondrocyte adhesion. The degradation cycle of the PLA scaffold was found to be consistent with the regeneration stage of cartilage defects, and in vivo results showed a good differentiation of the loaded chondrocytes in cartilage-like tissue [225]. The crystallisation in the α- or SC-phase of the PLA block depending on the initial materials was evidenced by different techniques; however, only the stereocomplexed materials were analysed in vivo [225].

##### Stents for Cardiac Regeneration

Stents are medical devices placed in a lumen of the body, particularly blood vessels, to prevent its closure. Metal stents are typically used to treat coronary disease due to their flexibility, radial force, resistance to fracture, radiopacity, biocompatibility, and low thrombogenicity. However, several late clinical complications including stent thrombosis, restenosis, and neoatherosclerosis still exist [18]. Bioresorbable vascular scaffolds emerged as a new technology in the field of percutaneous coronary intervention to provide temporary mechanical support and drug delivery, followed by bioresorption of the scaffold in the vessel. The first biodegradable stent in the clinical setting was developed 30 years ago and was constituted by PLLA [16]. Subsequently, several drug-eluting stents mainly based on PLLA and Rac-PLA were developed; particularly, BVS Absorb is widespread in clinical practice [238]. BVS Absorb was approved in 2016 by the FDA based on the non-inferiority test after 1 year of implantation compared to the corresponding “gold-standard”, namely, the metallic everolimus-eluting stent [239]. However, compared with metallic stents, the BVS appears to be associated with both a lower efficacy and a higher thrombotic risk after 2 years, on average [240], mainly due to scaffold discontinuity, malposition due to under-expansion, and neoatherosclerosis [241,242]. In addition, the current challenging generation of PLA-based scaffolds with radial strengths and flexibilities equivalent to their metallic stent counterparts [243] was recently addressed by investigating the influence of the stent geometric parameters on the mechanical properties. The radial stiffness and peak compression force of the PLLA helical stents were found to increase as both the initial pitch angle as well as the initial diameter decreased [244].

The physicochemical properties of PLA-based stents are rarely found (Table 5) despite having already reached clinical studies. In turn, it is difficult to replicate and improve the material performance from the issues encountered in the clinical step.

#### 6.2.2. Piezoelectric Activity

**Figure 8:**
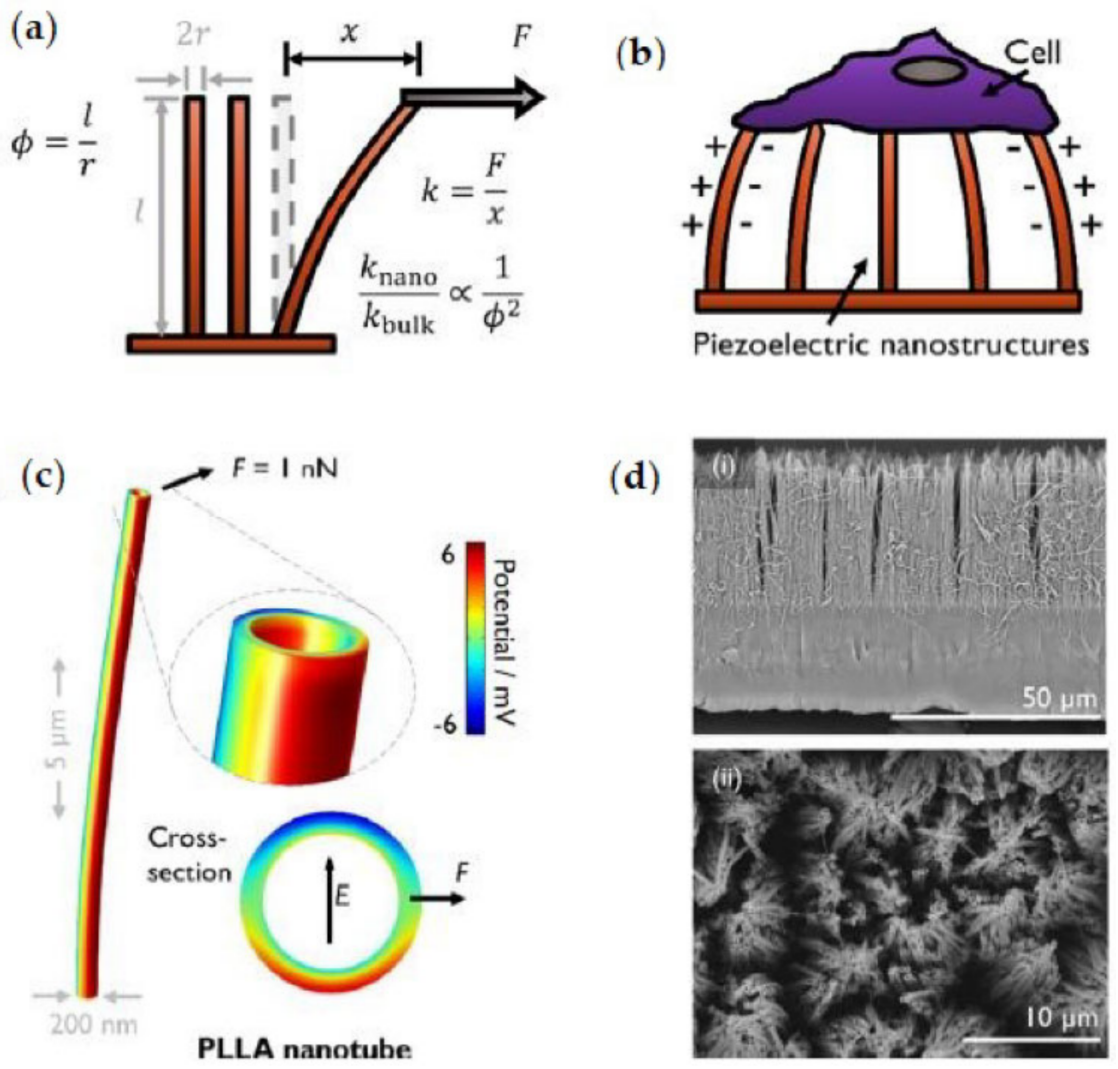
Piezoelectric activity of PLA. (**a**) Schematic of the bending mode of high-aspect-ratio structures with aspect ratio ϕ, which results in reduced effective stiffness by a factor of ϕ^−^^2^ with respect to the bulk material. (**b**) Proposed mechanism by which a cell can electromechanically stimulate itself by interacting with the piezoelectric nanostructures. (**c**) Simulation of a PLLA nanotube with axial polymer chain orientation, showing the potential developed in response to bending. Inset shows the opposing potentials developed across the tube diameter and the orientation of the corresponding electric field. (**d**) An example of the PLLA nanotube arrays produced via melt-press template wetting (**i**) in the cross-section and (**ii**) in the plan view. Adapted with permission from Ref. [251]. Copyright 2020, American Chemical Society.

The β-phase of PLA [45] as well as the oriented α form of PLLA films fabricated by solution casting and uniaxial stretching [246] exhibit piezoelectric characteristics (Figure 8) that could enhance the functional complexity designed for therapeutic applications. Indeed, the piezoelectricity of PLA has already been explored for its biomedical application as a film sensor device [247,248].

The voltage gradient present in cells can trigger different cell types to change proliferation and differentiation by signalling across membranes. The potential for harnessing the electric fields in cells to enhance growth and differentiation has recently gained attention. Likewise, the piezoelectric properties of PLA have been evaluated from a biomedical perspective, as the regeneration of damaged tissue starts with the growth and proliferation of cells [249]. Particularly, PLLA nanotubes produced via the melt-press template wetting technique provided a soft piezoelectric surface for biological studies in which a cell can electromechanically stimulate itself by interacting with the piezoelectric nanostructure [250]. Moreover, cell attachment could be regulated by controlling the crystallinity of PLLA nanotubes since the surface potential of the nanotubes increases subtly upon crystallisation [251].

Furthermore, a strong antibacterial effect due to the electric field generated by a piezoelectric PLLA yarn was recently observed, which could potentiate the applications of medical devices based on PLA [248].

#### 6.2.3. Shape Memory Polymers

**Figure 9:**
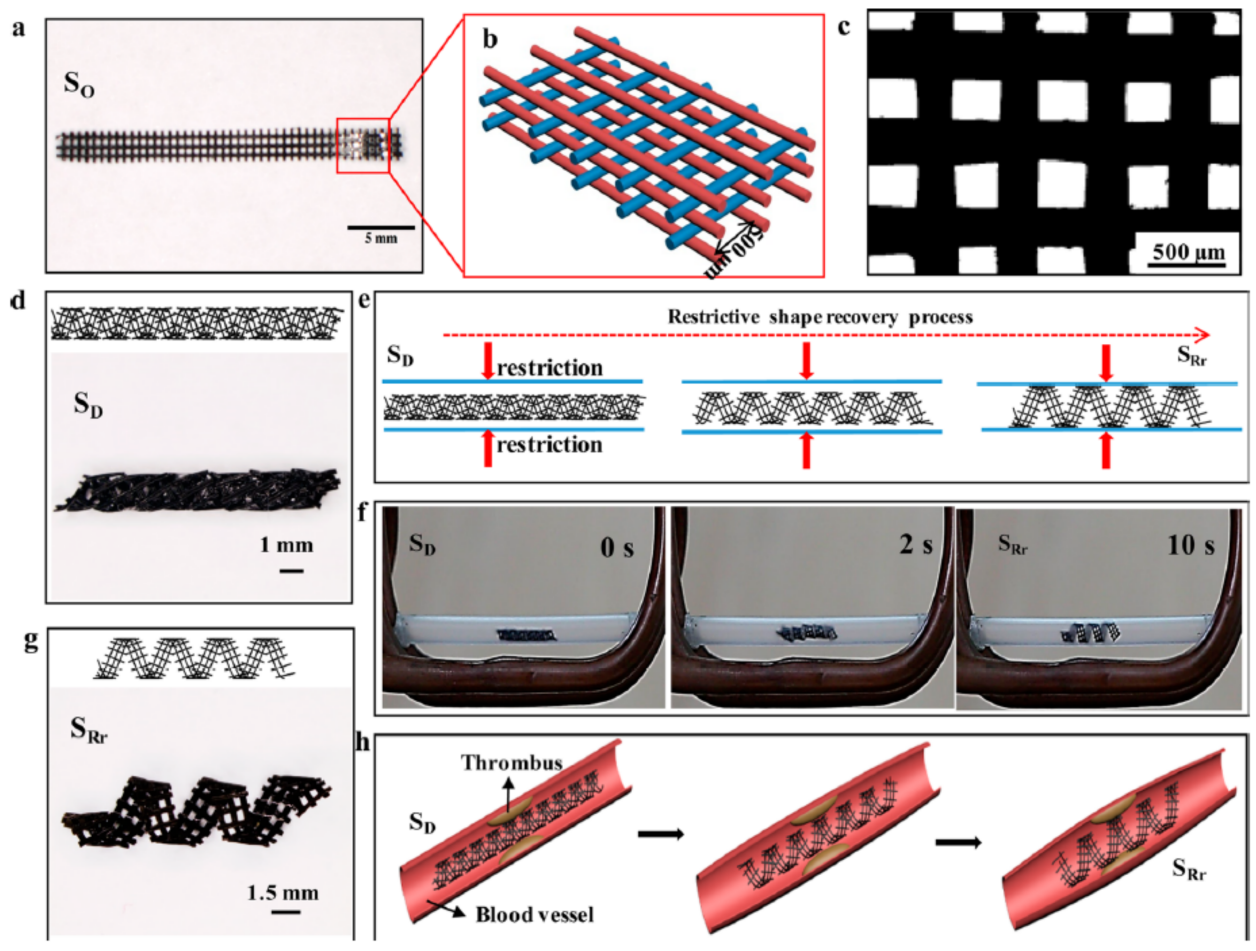
4D scaffold of c-PLA/Fe3O4 ink and its potential biomedical application. (**a**) Optical image of the multilayer scaffold; (**b**) schematic diagram of the detailed structure of the scaffold; (**c**) top view of the optical image of the printed scaffold; (**d**) deformation shape of the printed scaffold; (**e**) schematic of the restrictive shape recovery process; (**f**) demonstration of the restrictive shape recovery process triggered by a 30 kHz alternating magnetic field; (**g**) recovery shape under restrictive conditions; (**h**) potential application of the 4D scaffold as an intravascular stent. Reprinted with permission from Ref. [253]. Copyright 2017, American Chemical Society.

3D printing (3DP) is an additive manufacturing technology that allows for the mimicking of the complex and multicomponent structure of human tissues at reduced costs [85,86,89]. Moreover, the 4D printing processing method has emerged as an extension of 3D printing in which printed objects exhibit the capability to self-transform over time or in response to an external stimulus such as heat, light, pH, or magnetic and electric forces [252]. Likewise, shape memory polymers (SMP), after experiencing a shape deformation, are capable of recovering their initial shape upon an external stimulus [253] (Figure 9). Indeed, PLA has already been used as a bio-ink for 4D printing applications such as a spiral shape scaffold SMP generated from a PLA composite with magnetic iron oxide nanoparticles. Likewise, the iron oxide nanoparticles can be remotely heated and attracted by alternating magnetic fields, proving its application as a self-expandable stent with a minimally invasive intervention [253] as well as assisting in overcoming the lack of radial strength found for PLA stents compared to metallic stents. Similarly, porous tissue scaffolds attaining different internal structures formed from PLA with embedded magnetic nanoparticles have recently been developed by 3D printing, showing a great cell attachment and proliferation ability [254].

## 7. Conclusions

Polymers were envisaged as an alternative to conventional materials as well as to realise novel highly functionalised applications in the pharmaceutical and biomedical fields due to their ease in being shaped, lightness, industrial scalability, and chemical and biological inert nature. The current status of the cutting-edge applications in the pharmaceutical and biomedical fields based on PLA has been revised, covering the updates on the synthetic routes to attain multiblock PLA architectures and their structure–property relationships as well as the processing conditions impacting the performance of the final material. PLA is a bio-based polymer that has attracted significant interest due to its biodegradation capability and low price. PLA derivatives attaining different architectures are obtained depending on both the starting lactide derivative and the polymerisation method. The recently employed living polymerisation assisted by a single-site catalyst has enabled the attainment of PLA derivatives with high stereocontrol, high Mw, and narrow polydispersity, including stereo-block copolymers, which expands the availability of multiblock polymers on demand. In particular, high-Mw PLA stereo-block copolymers and their derivatives feature enhanced physicochemical properties that enlarge the potential biomedical and pharmaceutical applications. In addition, the rich PLA nanostructure, with diverse crystalline phases featuring different thermo-mechanical properties, as well as particular physicochemical properties such as piezoelectricity, are obtained through several processing techniques such as electro-spinning, 3D printing, or nanoparticle formulation. Likewise, the PLA crystalline domains degrade in physiological media at different rates depending on the crystalline PLA phase as well as the physicochemical and morphological parameters related to the surrounding medium, which must be considered when designing a biomedical device for a specific application.

Remarkably, PLA-based materials have already reached the clinical level in both pharmaceutical and biomedical applications such as sutures, stents, microparticles, and dermal fillers. In addition, PLA-based nanoparticles and 3D scaffolds are currently being developed for drug delivery and tissue regeneration applications, respectively. The piezoelectricity and chirality exhibited by PLA enlarge the functionalities and, in particular, the molecular recognition for potential biomedical devices. Likewise, the generation of bionanocomposites based on PLA affords the customisation of therapeutic materials with multifaceted capabilities. Furthermore, personalised therapeutic approaches have become feasible with the advent of 3D printing technology designing specific pharmaceutical formulations for specific patients, pointing toward personalised medicine.

However, the current gap between the PLA structural characterisation and the performance of PLA in biomedical applications hinders the systematic conception of personalised biomedical solutions based on the correlation of the structure–property relationship with the required functionalities. The end-user requirements should be considered from an interdisciplinary approach from the genesis of PLA-based material to realistically reach the pharmaceutical/biomedical market. Likewise, the PLA-nanoparticles formulation has greatly advanced to attain drug carriers down to nanoparticles with a fair control; however, the nanostructure development during the polymer processing is frequently dismissed, which is key to tuning the drug release profile as well as diminishing the batch-to-batch variability usually obtained through conventional techniques that hamper the scalability of nanoparticle formulation to the industrial scale. Moreover, the comprehensive nanostructure control achieved for a wide range of smart PLA-based hydrogels with specific functionalities must be followed by in vitro and in vivo studies to correlate the physicochemical properties with its performance under physiological conditions. In addition, the screening focus to develop new synthetic pathways to efficiently conjugate drugs to PLA should be also associated with a targeted PLA conjugate nanostructure in the applied medium in order to be translated reliably to both the release mechanism and the molecular recognition activity required to efficiently target the damaged tissue. Moreover, the tissue regeneration progress achieved from the materials approach requires a further understanding of the particular physicochemical and mechanical properties of each tissue, the alterations occurring under pathological conditions, and its evolution upon healing for designing materials according to the real requirements. Furthermore, the performance of the PLA-based stents suffers from structural stability due to the exhibited lack of radial strength that could be tacked by iterative structure-properties studies of novel multiblock copolymers. The mastering of novel polymer processing techniques, especially 3D techniques, should be achieved to generate customised materials with performing mechanical properties to attain consistently demanding applications.

## Figures and Tables

**Table 1:** Summary of the PLA-derivative nanoparticles.

Material	Processing Technique	Polymer Structure	Molecular Weight (kDa)	Material Characterisation ^1^	Studies ^2^	Ref.
Nanoparticles	Double emulsion-solvent evaporation	PLLA-PEG	85–160	Size, PDI, EE (%)	In vitro/in vivo	[149]
		PLLA				
	Nanoprecipitation-solvent displacement	Rac-PLA	-	Size, PDI, Z-potential, LE (%), EE (%), Morphology (SEM)	In vitro/in vivo/clinical	[150]
	Interfacial deposition-solvent displacement	Rac-PLA-Peg	18–28	Size, PDI, Z-potential	In vivo	[151]
	Emulsion-solvent evaporation	Maleimide-PEG-PLA (no PLA specification)	PEG_3_-PLA_70_	Size, Z-potential, Morphology (TEM)	In vivo	[152]
		Methoxy-PEG-PLA (no PLA specification)	PEG_3_-PLA_50_			
	Emulsion-solvent evaporation	PLGA (75L:25G)	15	CE (%) complexation efficiency, Z-potential, morphology (SEM)	In vivo	[153]
	Nanoprecipitation-solvent displacement	Trastuzumab-PEIcoating-Rac-PLA	22	LE (%), EE (%), Morphology (TEM)	In vitro	[154]
	Emulsion-solvent evaporation	Maleimide-PEG-PLA (no PLA specification)	PEG_3.4_–PLA_34_	Size, PDI, Z-potential, Morphology (TEM)	In vitro/In vivo	[155]
		Methoxy-PEG-PLA (no PLA specification)	PEG_3.4_–PLA_30_			
	Emulsion-solvent evaporation	Maleimide-PEG-PLA (no PLA specification)	PEG_3.4_–PLA_34_	Size, PDI, Z-potential, EE (%), LC (%), Morphology (TEM)	In vitro/In vivo	[156]
		Methoxy-PEG-PLA (no PLA specification)	PEG_3.4_–PLA_30_			
	Nanoprecipitation-solvent displacement	Rac-PLA-OH	10.5	Size, Z-potential, Morphology (TEM)	In vitro	[157]
		Rac-PLA-PEG-NH_2_	PLA_4.5_–PEG_3.5_–NH_2_			
	Nanoprecipitation-solvent evaporation	PLA	-	Size, Z-potential, pH, EE (%), LE (%), Morphology (AFM, SEM), Molecular structure (FTIR)	In vitro	[159]

^1^ After processing; ^2^ In vitro/in vivo/clinical.

**Table 2:** Summary of PLA-derivative hydrogels.

Material	Processing Technique	Polymer Structure	Molecular Weight (kDa)	Material Characterisation ^1^	Studies ^2^	Ref.
Hydrogels	Temperature increase of solutions (RT → 37 °C)	PLLA-Castor Oil	4.8	Viscosity, specific optical rotation, T_g_, shear stress	-	[162]
		Rac-PLA-Castor Oil	4.6			
	Temperature increase of solutions (RT → 37 °C)	PLLA	1.5	Sol-gel transition, micelles size, circular dichroism, morphology (TEM), nanostructure (XRD)	-	[169]
		Rac-PLA	1.5			
		PLLA-PEG	14.4			
		Rac-PLA-PEG	14.4			
	Hydrogelation by concentration	PLLA-PEG-PLLA	11.5–15.5	Storage modulus, nanostructure (WAXD)	-	[170]
		Rac-PLA-PEG-Rac-PLA				
	Temperature increase of solutions (RT → 37 °C)	OS-rac-PLA-PEG-rac-PLA-OS (olygomer serin)	~3	Sol-gel phase transition (depending on PEG M_w_)	In vitro/In vivo	[163]
	“Click” reaction	DA-NOCCL + N_3_ + rac-PLA-PEG-N_3_ (NP)	~6	Aggregates size, morphology (Crio-SEM and TEM), mechanical properties	In vitro/In vivo	[167]

^1^ After proccesing; ^2^ In vitro/in vivo/clinical.

**Table 3:** Summary of PLA-derivative polymer therapeutics.

Material	Processing Technique	Polymer Structure	Molecular Weight (kDa)	Material Characterisation ^1^	Studies ^2^	Ref.
Polymer therapeutics	ROP mediated by Paclitaxel	PEG-RacPLA-Ptxl	~30	Structure (NMR), M_w_ (GPC)	-	[206]
	ROP mediated by Docetaxel	PEG-RacPLA-Dtxl				
	Azide-alkine click reaction	Ptxl (23%)-RacPLA-PEG (25%)	~10–15	Size (DLS), structure (NMR), morphology (TEM), molecular weight (GPC)	In vitro	[207]
	Conjugation + micelles formulation	RacPLA-PEG-RacPLA-Lisinorpil	~10	Size (DLS), morphology (AFM)	In vitro drug release	[208]
	Hot press film	PLLA-indomethacin	-	Conjugation (fluorescence, XPS), contact angle	-	[209]
	ROP	(PLLA)_3_-Camptothecin [3-armed]	~30	Molecular weight (GPC), structure (NMR), morphology (AFM), thermal properties (TGA, DSC)	In vitro drug release and enzymatic degradation	[210]

^1^ After processing; ^2^ In vitro/in vivo/clinical.

**Table 4:** Summary of PLA-derivative scaffolds.

Material	Processing Technique	Polymer Structure	Molecular Weight (kDa)	Material Characterisation ^1^	Studies ^2^	Ref.
Scaffolds	Freeze-drying, salt-leaching (3D scaffold)	PLGA (75L:25G) + Silk + HA(Hidroxiapatite)	90–126	Structure (FTIR), degradation temperature (TGA), morphology (SEM), swelling (%), water uptake (%), mechanical properties	In vitro/In vivo	[214]
	Wet-spinning (microfilament)	P(L-co-rac-LA) (75:25)	200	Morphology (SEM)	In vivo	[215]
	Bought (kinnet filmanets)	PGA/PLA (2:1)	-	Morphology (SEM and TEM), biomechanical properties	In vitro/In vivo	[216]
	Bought (forged composite sheets)	PLLA-PGA (88:12) + HA	-	Morphology (SEM)	In vivo	[217]
		PLLA + HA				
	Bought (OSTEOTRANS MX)	PLLA + HA	-	Molecular weight, crystallinity, morphology (SEM) (after surgery)	Clinical	[218]
	Bought (plates)	PLLA/PGA	-	-	Clinical	[219]
		PLLA/HA				
	Electrospinning	PLGA (85/15)	285	Morphology (SEM), mechanical properties, structure (FTIR)	In vitro	[220]
	Electrospinning	PLGA (85/15)	285	Morphology (SEM), mechanical properties, structure (FTIR)	In vitro	[221]
	Supercritical emulsion extraction	PLGA carriers + fibrin hydrogel	38–54	Size, morphology (SEM)	In vitro	[222]
	Electrospinning	PLGA + silk	-	Morphology (SEM), mechanical properties	In vitro	[223]
	Knitted fibres	PLGA (10/90)	-	Mechanical properties	In vivo	[224]
	Micelles in an aqueous solution (hydrogel)	Four-armed PEG-(PLLA)_4_	PEG_10_-PLLA_1_	Size, morphology (TEM), nanostructure (FTIR, XRD), thermal properties (DSC), mechanical properties	In vitro	[225]
		Four-armed PEG-(PDLA)_4_	PEG_10_-PDLA_1_			
		Four-armed PEG-(PLLA-Cho)_4_	PEG_10_-PLLA_1_-Chol			
		Four-armed PEG-(PDLA-Chol)_4_	PEG_10_-PDLA_1_-Chol			
	Hydrogel by solution	PDLA-PLLA-PEG-PLLA-PDLA	~20	Specific optical rotation, gel-sol transition, physical gelation, nanostructure (WAXS), microstructure (SAXS)	In vitro drug release	[164]

^1^ After processing; ^2^ In vitro/in vivo/clinical.

**Table 5:** Summary of PLA-derivative stents.

Material	Processing Technique	Polymer Structure	Molecular Weight (kDa)	Material Characterisation ^1^	Studies ^2^	Ref.
Stents	Bought (REMEDY) ^3^	Rac-PLA	-	-	Clinical	[16]
	Desolve Cx ^3^	PLLA-Novolimus	-	In vitro/in vivo degradation (M_W_ lost)	Clinical	[17]
	Mirage ^3^	PLLA (4% D-LA)-sirolimus	-	-	Clinical	[18]
	ABSORB V G2 ^3^	PLLA	-	-	Clinical trial (RENASCENT III)	[245]
	MAGNITUDE ^3^	PLLA-sirolimus	-	-	Clinical	[19]

^1^ After processing; ^2^ In vitro/in vivo/clinical; ^3^ Commercial name of the stent.

## Abbreviations

3DP	Three-dimensional printing
DDS	Drug delivery system
D-LA	D-Lactide
FDA	Food and Drug Administration
HC	Homocrystals
HM_w_	High molecular weight
i-PP	Isotactic polypropylene
k_h_	Hydrolysis constant
k_H_	Degradation constant catalysed by protons
k_o_	Degradation constant in water
k_OH_	Degradation constant catalysed by hydroxyl ions
LA	Lactide
L-LA	L-Lactide
M_w_	Molecular weight
PCL	Poly(e-caprolactone)
PCM	Polymer of chiral acetylenic
PDLA	Poly(D-lactide)
PE	Polyethylene
PEG	Polyethylene glycol
PET	Polyethylene terephthalate
PEVA	Poly(ethylene-*co*-vinyl acetate)
PGA	Polyglycolic acid
PHA	Polyhydroxhyalkanoate
PLA	Polylactic acid
PLGA	Poly(lactic-*co*-glycolic) acid
PLLA	Poly(L-lactide)
PMMA	Poly(methyl methacrylate)
PP	Polypropylene
PS	Polystytene
Rac-LA	Racemic-lactide
ROP	Ring-opening polymerisation
SC	Stereocomplex
SMP	Shape memory polymer
T_c_	Crystallisation temperature
TE	Tissue engineering
T_g_	Glass transition temperature
T_h_	Hydrolytic degradation temperature
T_m_	Melting temperature
X_c_	Crystallinity
